# Iron-Catalyzed Oxidative Coupling under Aerobic Conditions: A Decade of Progress

**DOI:** 10.1021/acsomega.6c01942

**Published:** 2026-06-08

**Authors:** Charan D.C., Navami Prabhu, Suresh D. Kulkarni, Nitinkumar S. Shetty

**Affiliations:** † Manipal Institute of Technology, 76793 Manipal Academy of Higher Education, Manipal 576104, India; ‡ Manipal Institute of Applied Physics, Manipal Academy of Higher Education, Manipal 576104, India

## Abstract

The progressive maturation of oxidative transformations is perhaps most compellingly reflected in the selectivity of iron-catalyzed homonuclear C–C and C–X linkages. With recent developments contextualized within the existing literature, this review offers a rich resource for those focused on efficient and sustainable methodologies. This review focuses on homogeneous, heterogeneous, and cooperative catalytic systems, ligand-engineered iron complexes, and environmentally conscious reaction parameters. Strategic catalyst modulation and the scope of reaction pathways are analyzed in depth, highlighting both substrate versatility and the robustness of the system, while providing a roadmap for future innovation and addressing existing limitations.

## Graphical abstract

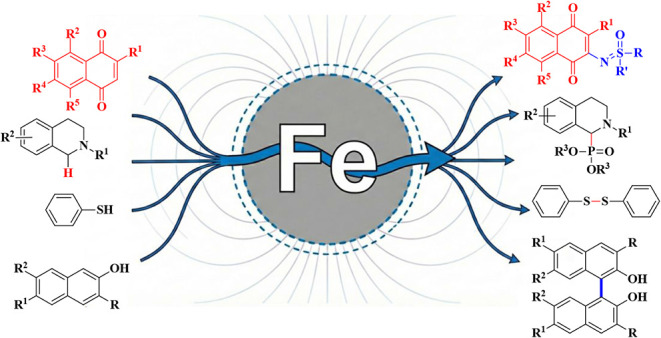

## 1 Introduction

The landscape of contemporary organic synthesis is increasingly defined by the strategic deployment of oxidative coupling protocols that facilitate the direct assembly of carbon–carbon and carbon–heteroatom linkages. By circumventing the traditional necessity for prefunctionalized precursors and the requirement for protecting groups, these methodologies enable a streamlined approach to molecular complexity.^1,2^ Such transformations find purpose across a myriad of synthetic frontiers, from the total synthesis of natural products^3^ to the rigorous demands of pharmaceutical development.^4,5^ The capacity to fabricate densely functionalized frameworks^6^ and multifunctional heterocycles^7,8^ with high atom economy renders this approach an indispensable instrument for both academic inquiry and industrial applications.

The genesis of this discipline traces back to the mid-19^th^ century, particularly with the pioneering investigation of Julius Löwe (1868) regarding the silver oxide- or arsenic acid-promoted oxidation of gallic acid to furnish ellagic acid^9^ and the preparation of BINOLs (1,1′-bi-2-naphthols) by Alexander Dianin (1873).^10^ Although these early studies laid the foundation for the principles of oxidative dimerization, they preceded the development of catalytic systems, as stoichiometric amounts of oxidants were used instead. Traditional methods have typically employed a wide variety of reagents, including chromium^11^ and manganese reagents,^12,13^ hypervalent iodine reagents,^14,15^ and copper^16^ and silver salts,^17^ which resulted in large amounts of waste and were carried out under harsh conditions that are not compatible with sensitive functional groups.^18,19^ The toxicity of these reagents, in addition to the inefficiency of using prefunctionalized substrates,^20^ often posed a problem in the formation of complex targets, which led to the transition toward catalytic oxidative coupling. By overcoming these harsh constraints, modern strategies now enable direct activation under refined and selective conditions, which greatly enhances efficiency while minimizing the environmental impact of bond formation.^21^

A major paradigm shift from stoichiometric coupling methods came in 1959 with the introduction of a catalytic method by Hay using copper­(I) chloride and molecular oxygen.^22^ This historic achievement, which formed the basis for poly­(phenylene)­oxide synthesis and the Glaser-Hay coupling, laid the guiding principles of modern aerobic catalysis. Redox catalytic strategies have since progressed from reagent-wasteful stoichiometric methods to transition-metal-catalyzed bond-forming events through carefully controlled activation cycles. Today, copper catalysts allow aerobic C–N^23^ and C–O^16^ coupling, while palladium complexes mediate oxygen-driven dehydrogenation reactions.^24^ Moreover, nickel and cobalt catalysts facilitate C–H activation pathways that do not require substrate prefunctionalization.^25,26^ To complement these metal-catalyzed systems, organic catalysts such as nitroxyl radical derivatives of 2,2,6,6-tetramethylpiperidine-1-oxyl (TEMPO) facilitate aerobic coupling reactions without resorting to toxic additives.^27^ These developments have together minimized waste and generated a synthetic platform characterized by increased chemoselectivity and compatibility with a wide range of functional groups.

The pursuit of catalytic systems that use minimal reagent load has led to a focus on aerobic oxidative coupling. Molecular oxygen (O_2_) is particularly noteworthy for its high atom economy and low waste production compared to stoichiometric reagents.^28^ Nevertheless, the key to successful aerobic coupling is the use of catalysts that can overcome the spin barrier imposed by the triplet state of O_2_, which makes direct reactivity with organic substrates kinetically unfavorable.^29^ These catalysts facilitate electron transfer to generate superoxo, peroxo, or high-valent metal-oxo intermediates,^30^ whose reactivity is responsible for bond formation via hydrogen atom abstraction or radical-mediated mechanisms.^31–33^ In this context, iron-based catalysts occupy a unique position because of their natural abundance in the Earth’s crust, nontoxicity, and diverse redox properties that include Fe­(II), Fe­(III), and high-valent ferryl species.^34^ These oxidation states provide selective reactivity that mirrors the heme and nonheme oxygenase enzymes.^35^ By paralleling these natural models, iron-based catalysts provide a versatile, sustainable, and scientifically sound platform for aerobic oxidative coupling.

In this comprehensive review, we provide a critical assessment of the literature over the last decade (2015–2025) and highlight the key developments in iron-catalyzed aerobic oxidative coupling. Our primary goal is to identify the approaches that integrate the principles of sustainability with the practical aspects of synthesis. Ultimately, we aim to provide a roadmap that addresses the increasing need for environmentally benign molecular synthesis, while providing readers with a comprehensive overview of approaches utilizing iron catalysts and emphasizing their transformative impact on the development of green organic synthesis.

## 2 Fundamentals and Reactivity Principles of Aerobic Oxidative Coupling

### 2.1 Coordination Environment and Electronic Properties of Iron

Iron’s reactivity in oxidative processes is governed by its electronic configuration and how the ligand field splits and orders the *d* orbitals at the metal center. Variations in donor strength, denticity, and geometric constraints control this splitting and tune the electronic distribution, giving rise to distinct spin states that modulate reactivity.^36^ Strong-field, octahedral Fe­(II) complexes such as low-spin polypyridyl^37^ or *N*-heterocyclic carbene^38^ species typically display a spin quantum number *S* = 0 (t_2g_)^6^ configuration that prefers more inert, inner-sphere electron transfer, whereas weaker-field or distorted octahedral environments often generate high-spin *S* = 2 Fe­(II) centers with enhanced lability and higher oxidative reactivity. Relatedly, trigonal-bipyramidal high-spin *S* = 2 Fe­(IV)-oxo complexes are documented to facilitate C–H activation at rates that substantially exceed those of *S* = 1 species, directly demonstrating how spin states control oxidizing power.^39,40^ Octahedral, square-pyramidal, and trigonal-bipyramidal coordination geometries are frequently encountered in synthetic nonheme iron systems. Among these, modest structural distortions—such as trigonal twisting or the enforcement of a trigonal-bipyramidal environment—attenuate the ligand field strength, thereby favoring high-spin configurations and consequently enhancing the capacity of the iron center to access multiple oxidation states and engage in substrate and O_2_ activation.^41,42^

These features dictate the manner in which iron facilitates electron transfer via inner- and outer-coordination spheres, its involvement in proton-coupled electron transfer, and its accommodation of shifting electronic topologies during oxidative transformations. High-spin, more labile centers can more easily access multiple oxidation states and transient peroxo or oxo intermediates, whereas low-spin, more rigid complexes favor controlled, stepwise redox cycling with well-defined potentials. Spectroscopic techniques such as Mössbauer spectroscopy,^43^ EPR,^44^ and resonance Raman^45^ have provided extensive insight into these factors by directly probing the formal oxidation level, spin multiplicity, and spatial geometry of the iron atom during catalysis and model reactions, thereby linking ligand field and coordination geometry to observed reactivity.

### 2.2 Role of Molecular Oxygen in Aerobic Oxidative Coupling

Molecular oxygen functions as the terminal oxidant in aerobic oxidative coupling by accepting electrons generated during the substrate transformation. In catalytic systems employing iron, O_2_ undergoes stepwise reduction at the metal center, enabling controlled oxidation of organic substrates without the accumulation of hazardous byproducts.^46^ This reliance on oxygen circumvents the need for stoichiometric oxidants, such as permanganates, chromates, or hypervalent iodine reagents, thereby reducing reagent consumption and improving overall atom efficiency.^47^ The environmentally favorable nature of O_2_ stems from its benign reduction product, water, which simplifies workup and minimizes aqueous waste streams.^48^ An effective catalyst accelerates the otherwise spin-forbidden interaction between triplet oxygen and closed-shell organic molecules, and iron complexes are well-suited for this because of their accessible redox states and ability to coordinate and activate O_2_ under mild conditions.^35^

### 2.3 Mechanistic Features of Iron-Catalyzed Aerobic Oxidative Coupling

In aerobic oxidative coupling, iron catalysts typically operate through a redox cycle in which an Fe­(II) species is oxidized to an Fe­(III) species by molecular oxygen.^49^ During this step, reactive oxygen species (ROS) such as superoxo- or peroxo-type intermediates are generated, and these species are responsible for initiating substrate activation.^50^ The ROS facilitate hydrogen abstraction from the substrate, yielding radical precursors, and these species then engage in radical recombination or coupling with secondary radical species to furnish the coupled product. After the bond-forming step, the iron center returns to its lower oxidation state, allowing the catalyst to turn over repeatedly under an O_2_ atmosphere.^51^

In certain reported systems, halogen promoters such as I_2_ are employed in conjunction with iron catalysts, wherein transient iodophosphate intermediates are generated in situ, and the Fe/I manifold facilitates turnover under molecular oxygen as the terminal oxidant.^52^ In addition to the catalyst design, operational parameters, including temperature, solvent, and oxygen pressure, exert a profound influence on the rate and outcome of the process; therefore, their optimization is essential for high yields and good selectivity. Substrate electronic properties also play an important role, with electron-rich substrates generally reacting more readily than electron-poor analogues.^53^

## 3 Iron Catalysts in Aerobic Oxidative Coupling

### 3.1 Iron Salt-Based Catalytic Systems

For the first time, Sharma *et al.* disclosed a visible-light-driven strategy for accessing naphthoquinone-sulfoximine derivatives, establishing simple FeCl_3_ as a highly effective catalyst in facilitating this conversion.^54^ Notably, FeCl_3_ outperformed common organic photocatalysts such as Eosin Y and Rose Bengal, which delivered only moderate yields of ***(1a)*** and increased by-product formation, underscoring the intrinsic photoactivity of the iron system rather than a conventional photoredox pathway. The lack of yield enhancement upon coordination with 1,10-phenanthroline (Phen) or 2,2′-bis­(diphenylphosphino)-1,1′-binaphthyl (BINAP) further suggests that the active species operates through a minimal iron manifold. Conducted in ethanol at room temperature, the protocol represents a genuinely green and atom-efficient transformation. Its successful extension to complex naphthoquinones, including Vitamin K3 and Juglone, highlights the method’s synthetic robustness and potential relevance for modification of bioactive compounds (Scheme 1).

**Scheme 1:**
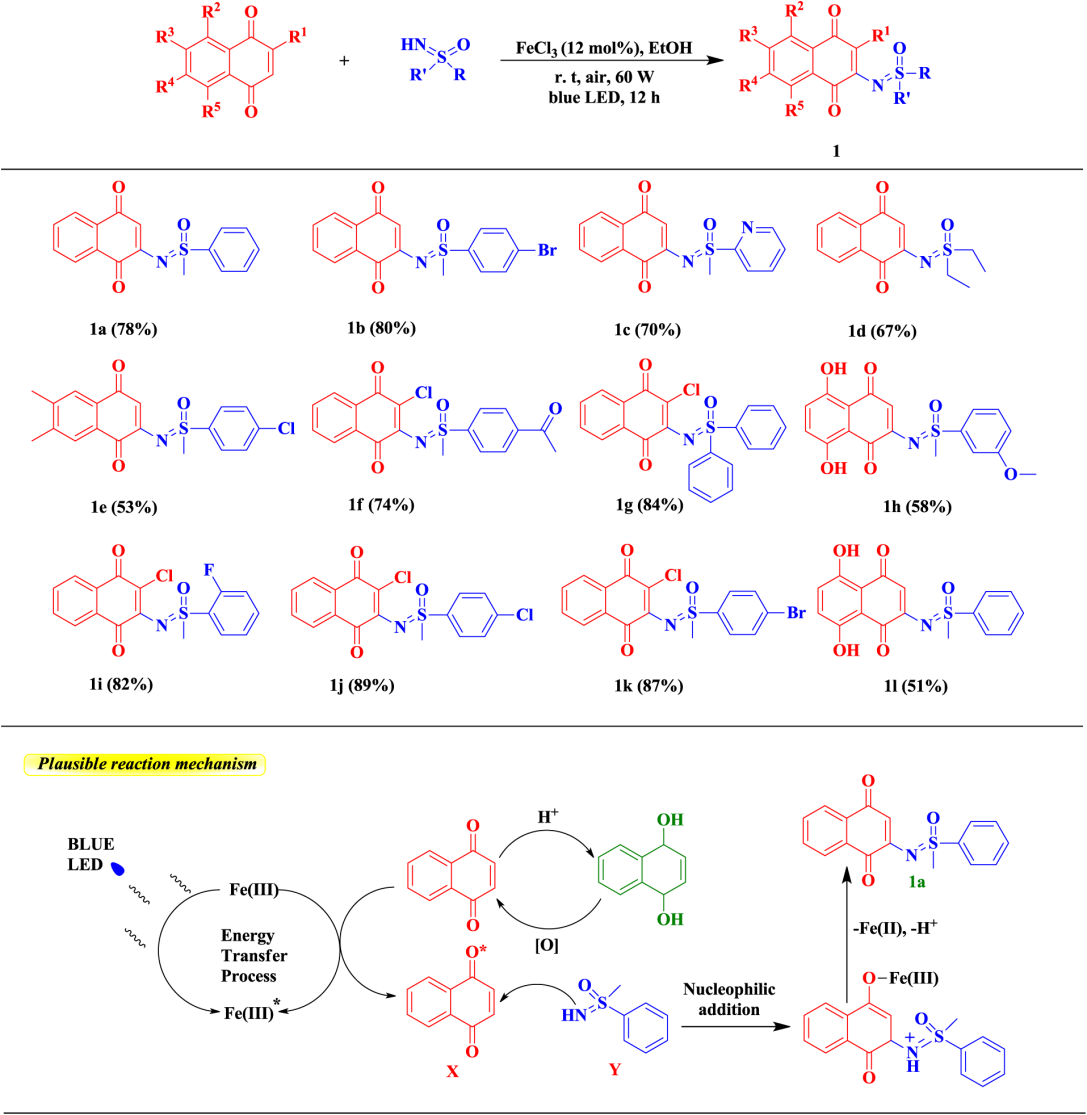
FeCl_3_-Catalyzed Visible-Light-Promoted Oxidative C–N Coupling of 1,4-Naphthoquinones with Sulfoximine

An FeCl_3_-photocatalyzed oxidative amidation of benzylic C–H bonds, enabling direct access to amides (Scheme 2) and fused nitrogen-containing heterocycles (Scheme 3), was introduced by Yang and colleagues.^55^ A key insight of the study is the deliberate use of a photoinduced iron-ligand charge transfer (Fe-LMCT) process to generate reactive chlorine radicals in situ. The observed enhancement of added chlorine and formation of chlorinated styrene adducts provide compelling evidence that chlorine radicals act as hydrogen atom abstractors, initiating C–H activation. Mechanistic investigations revealed that the substitution of FeCl_3_ with Fe­(OTf)_3_/AcCl still afforded the amidated product, indicating that AcCl serves as the primary chlorine-radical source, and the complete suppression of reactivity in its absence confirmed the requirement for a chlorine-oxygen donor in the photoinduced Fe-LMCT pathway, while ^18^O labeling experiments verified incorporation of the labeled oxygen atom into the amide product by HRMS.

**Scheme 2:**
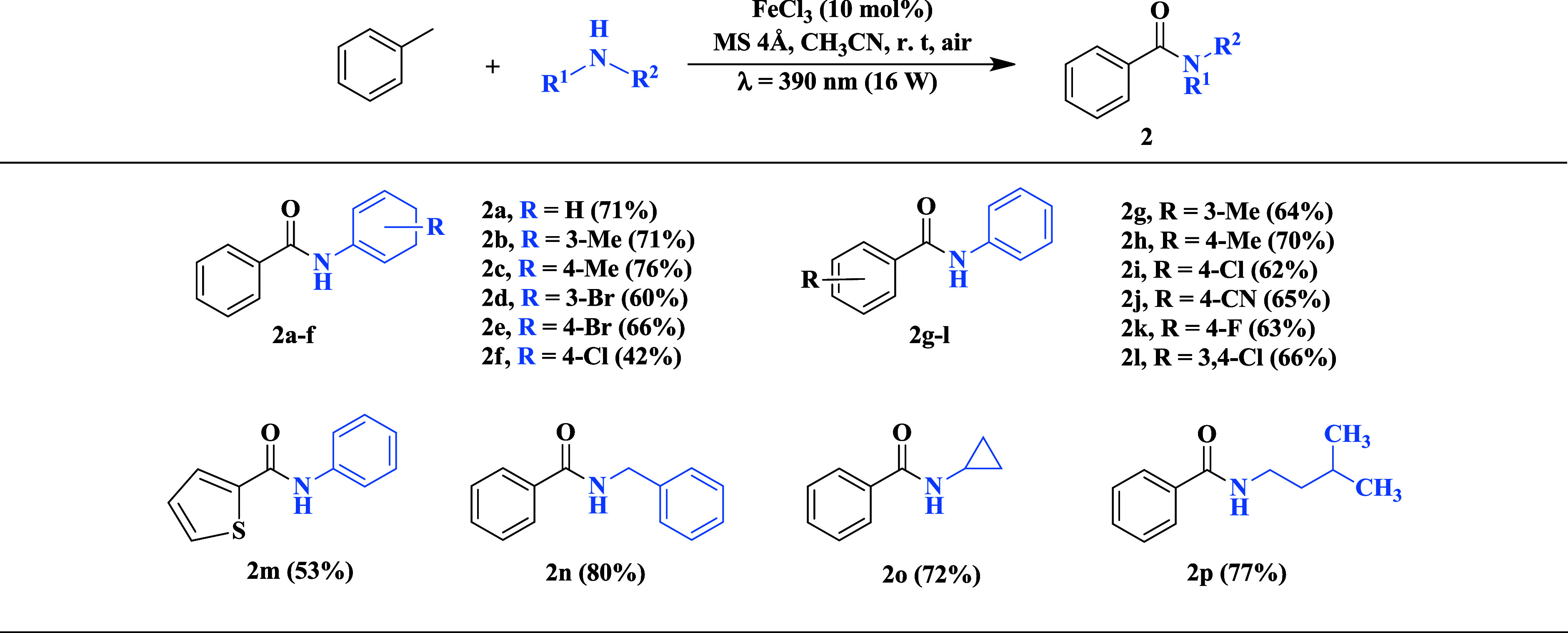
FeCl_3_-Catalyzed Benzylic C–H Oxidative Amidation of Amines

**Scheme 3:**
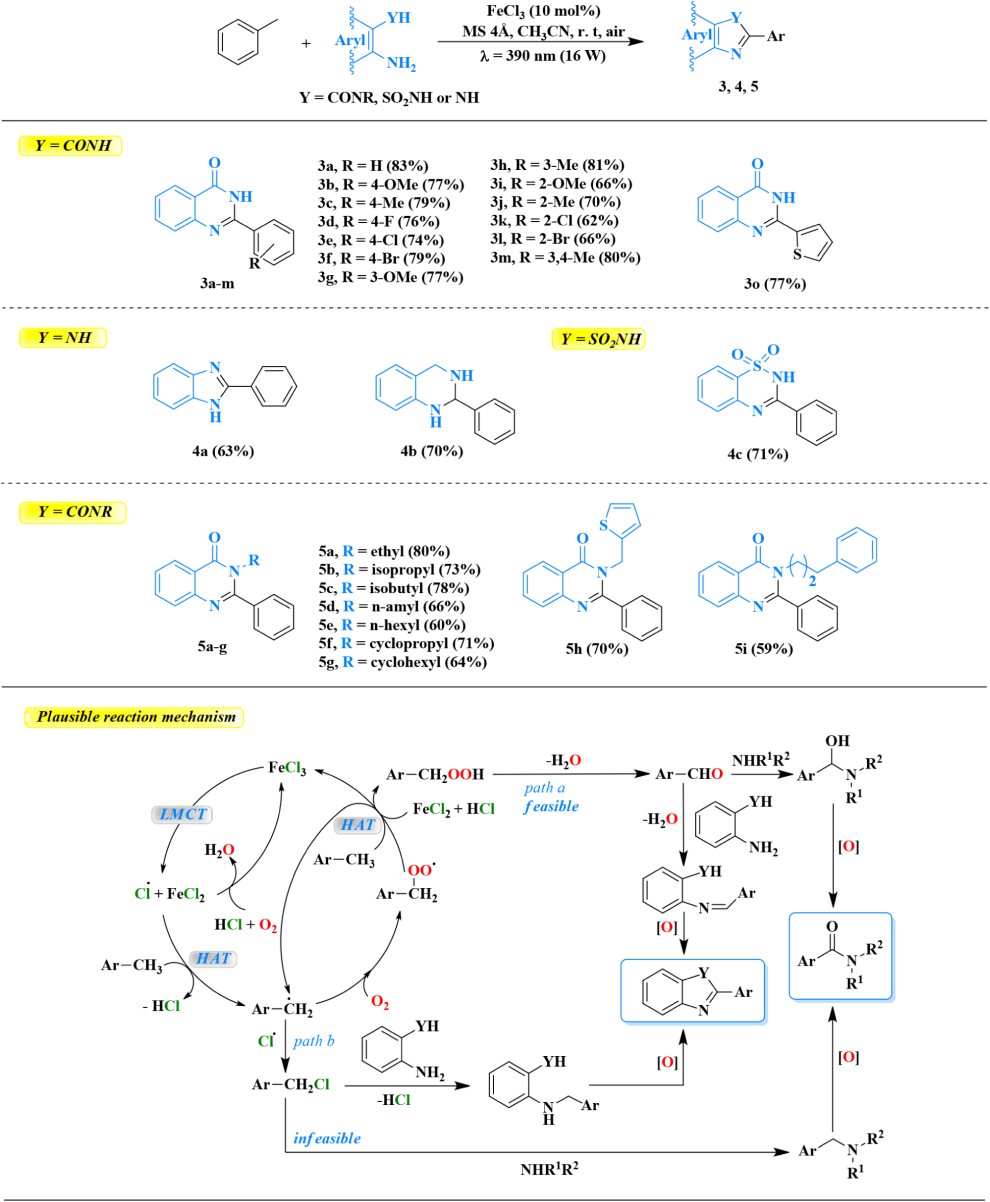
FeCl_3_-Catalyzed Benzylic C–H Oxidative Amidation of *o*-Amino Arylamides

Singh and Prasad^56^ reported a one-pot, iron-catalyzed cross-dehydrogenative coupling (CDC) strategy for the synthesis of α, β-unsaturated ketones through direct C­(*sp* ^3^)–H functionalization of methylarenes with acetophenones (Scheme 4). The transformation is highly sensitive to the reaction medium, proceeding efficiently only in polar aprotic solvents such as DMSO and DMF, while protic and nonpolar solvents fail to sustain productive turnover. Increasing FeCl_3_·6H_2_O loading beyond 10 mol % offers no measurable benefit. Suppression of product formation by TEMPO and under inert conditions establishes the involvement of radical intermediates and the necessity of molecular oxygen. The protocol enables direct access to chalcones, including those related to the choleretic agent Metochalcone, reinforcing the utility of iron for assembling bioactive enone motifs.

**Scheme 4:**
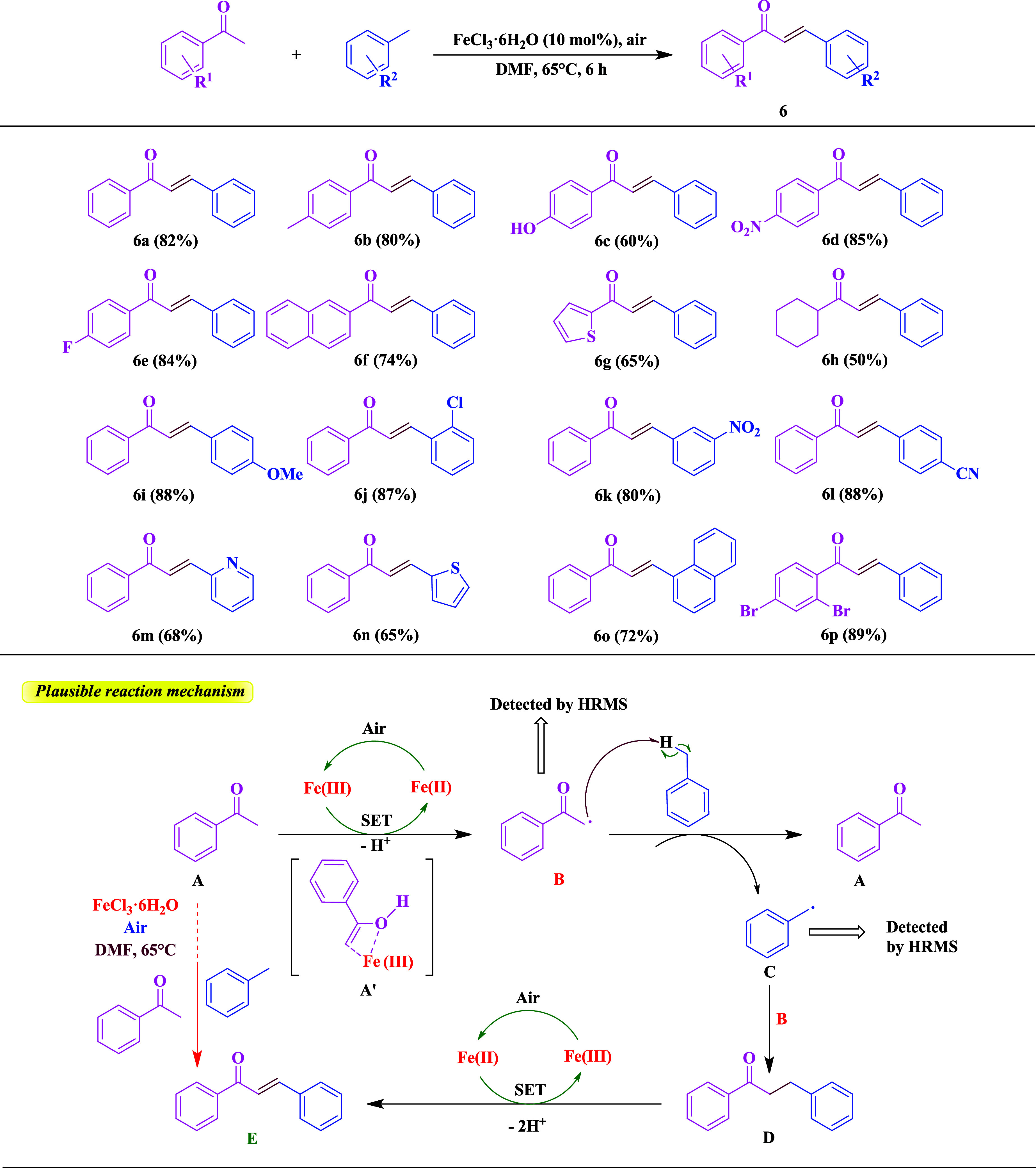
Synthesis of α, β-Unsaturated Ketones through FeCl_3_-Mediated Oxidative CDC

The oxidative union of indolin-2-ones with indoles, examined by Wu *et al*.,^57^ generated 3,3-disubstituted oxindoles bearing quaternary carbon centers from C­(*sp* ^3^)–H precursors (Scheme 5).

**Scheme 5:**
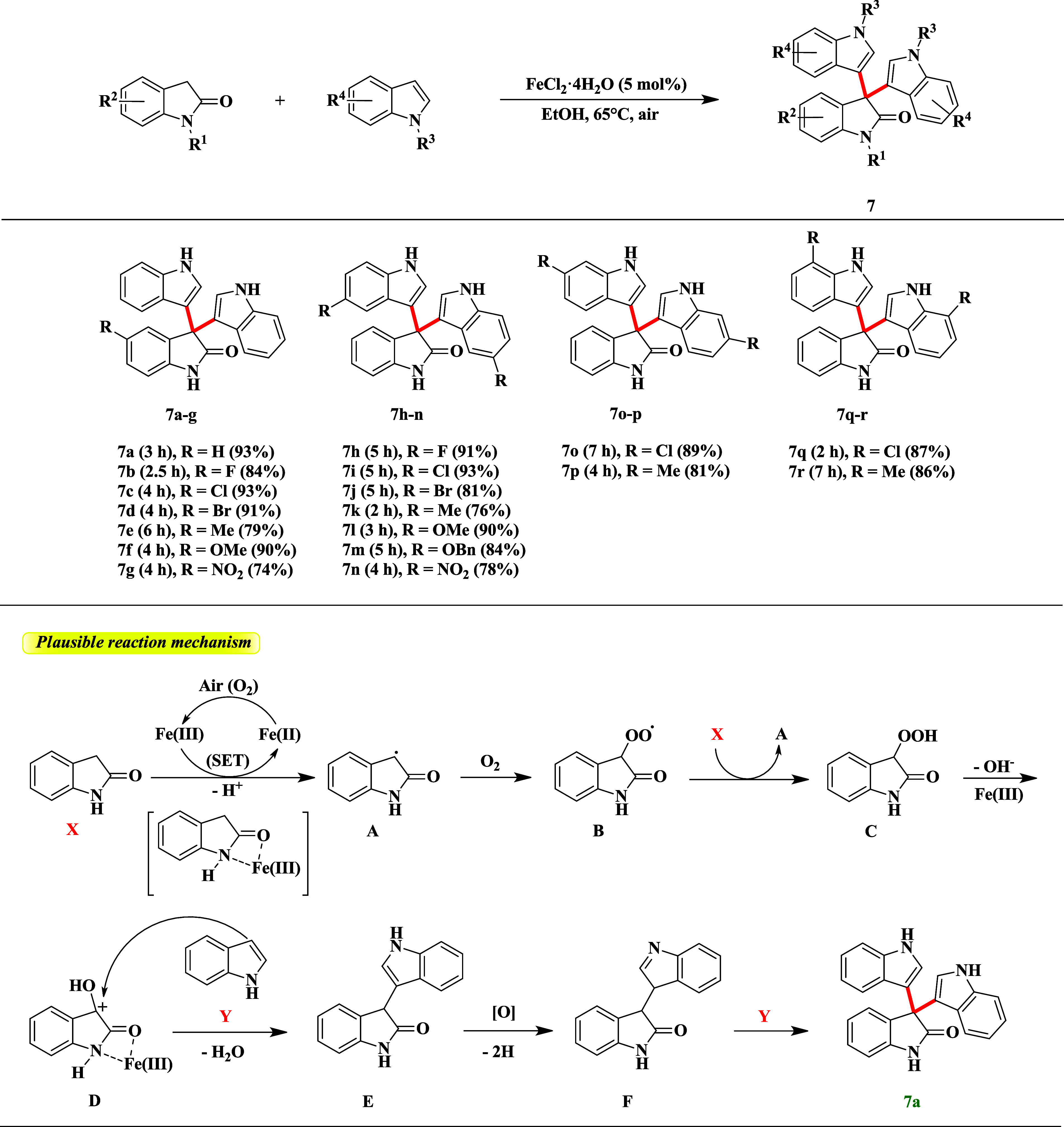
FeCl_2_·4H_2_O-Catalyzed Oxidative Bis-Arylation of Indolin-2 Ones

Comparative evaluation of iron and other transition metal salts, including FeBr_3_, Cu­(OAc)_2_, NiCl_2_, and Yb­(OTf)_3_, revealed that FeCl_2_·4H_2_O provides a redox environment well-suited for controlled C–C bond formation. The transformation proceeds efficiently across indolin-2-ones substituted at the C-4, C-5, C-6, and C-7 positions, irrespective of electronic bias, indicating limited sensitivity to substitution patterns. The transformation remained compatible with *N-*protected substrates and strongly deactivating groups such as NO_2_. Successful gram-scale preparation of trisindoline and a recognized spermicidal compound reinforces the operational scalability of this strategy.

Documented by Wu and coworkers,^58^ an Fe­(III)-catalyzed strategy enables the synthesis of 3,3′-disubstituted oxindoles through direct cross-dehydrogenative coupling of 3-substituted oxindoles with electron-rich arenes (Schemes 6, 7). The preference for FeBr_3_ under aerobic conditions suggests that both Lewis acidity and halide identity finely modulate radical formation and intermediate stability. Trends in C-3-substituted oxindoles revealed that the nature of the C-3 substituent primarily dictates the efficiency of radical formation, rather than the coupling event itself. The observed regioselectivity with activated arenes reflects a sequence in which single-electron oxidation generates a radical intermediate that, upon further electron transfer, engages the arene through an electrophilic-type coupling. The use of a chlorinated solvent for electron-rich arenes further highlights solvent-mediated modulation of arene activity.

**Scheme 6:**
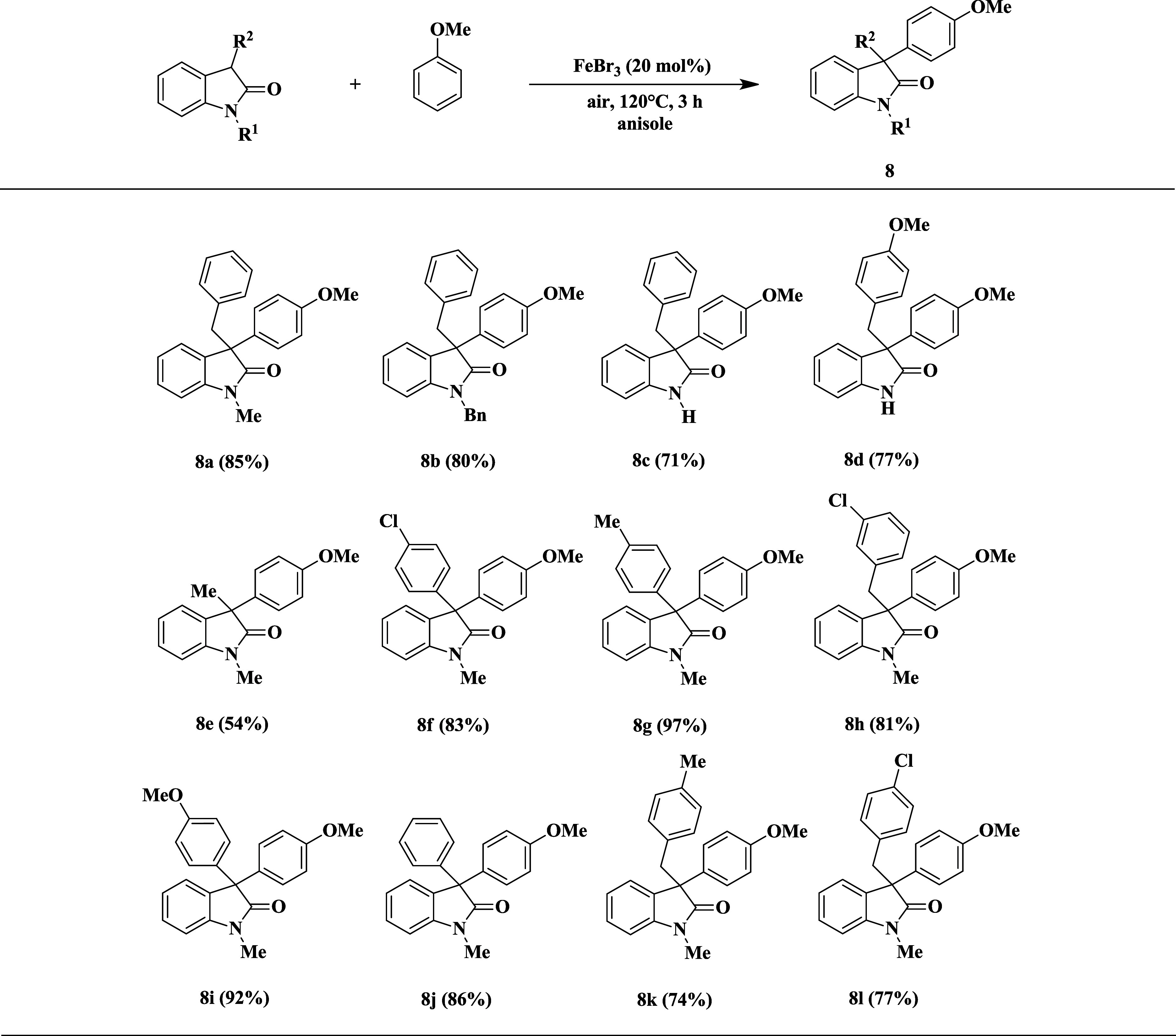
FeBr_3_-Catalyzed Oxidative Cross-Dehydrogenative Arylation between Oxindoles and Anisole

**Scheme 7:**
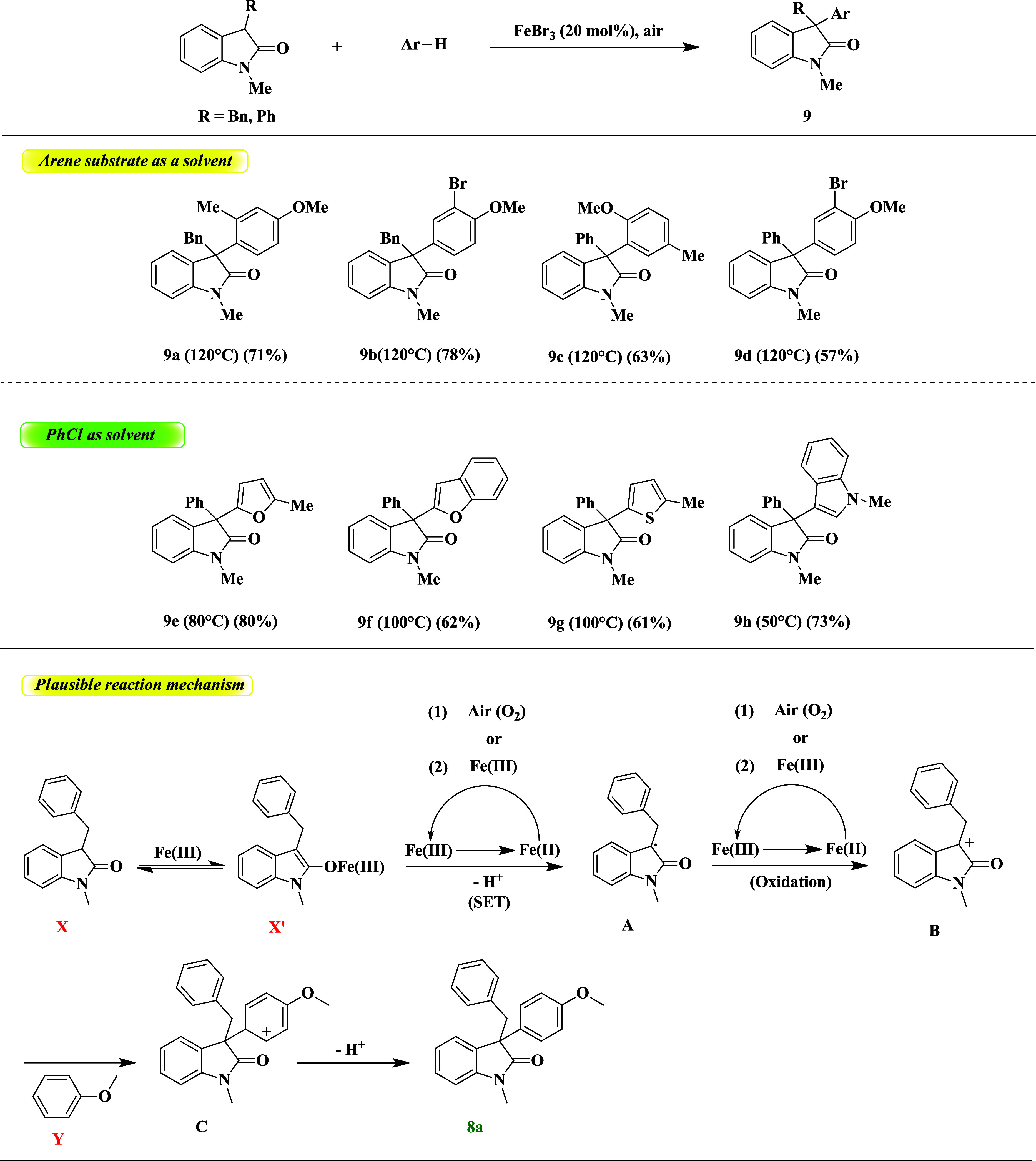
FeBr_3_-Catalyzed Oxidative Cross-Dehydrogenative Arylation between Oxindoles and Arenes

Kumar *et al.* ^59^ showcased how simple iron salts can promote direct C–N bond formation between 2-aminobenzothiazoles and primary amines under aerobic conditions, with FeBr_2_ providing a well-balanced catalytic profile (Scheme 8). The sharp dependence on catalyst loading and the near-complete shutdown under an inert atmosphere indicated a tightly controlled oxidative turnover reliant on atmospheric oxygen. Differences in reactivity between benzylic, aliphatic, and heteroaryl amines suggest that hydrogen atom abstraction efficiency, rather than amine nucleophilicity, governs productive coupling.

**Scheme 8:**
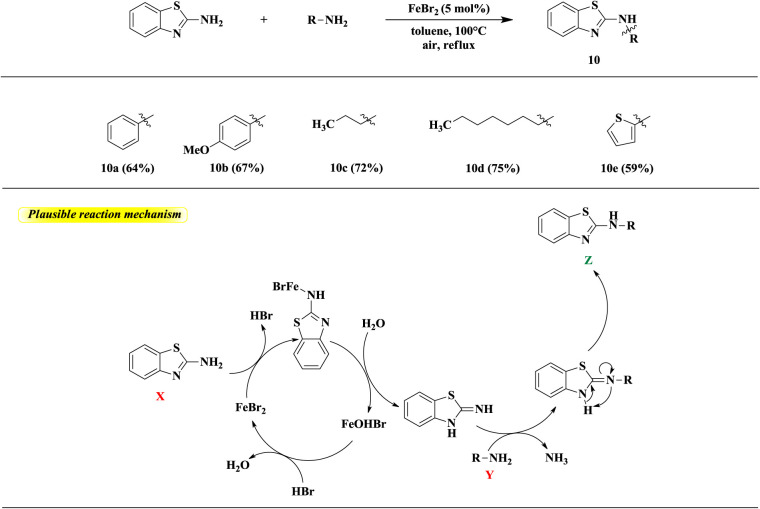
FeBr_2_-Catalyzed Oxidative Coupling for Synthesis of *N*-Substituted 2-Aminobenzothiazoles

A highly efficient iron-catalyzed aerobic oxidative phosphonation, developed by Cai *et al.*,^60^ facilitates the synthesis of α-aminophosphonates, which serve as important structural motifs and bioisosteres of α-amino acids with high potential as enzyme inhibitors. The combination of Fe­(NO_3_)_3_·9H_2_O and air under mild, open-flask conditions demonstrates that inexpensive iron salts can promote C–P bond formation with operational simplicity. The reaction efficiently couples various dialkyl phosphites and *N-*aryltetrahydroisoquinolines, highlighting substrate tolerance and complementary reactivity between the coupling partners (Scheme 9). Mechanistic insights supported by ESI-MS monitoring indicate the formation of iminium intermediates and related derivatives, while TEMPO inhibition studies revealed that the radical quenching effect is secondary to coordination-induced deactivation confirmed by detection of the [(TEMPO)­Fe­(NO_3_)_3_ + K^+^] adduct (m/z = 437.3). These observations collectively suggest a synergistic interplay between the Fe­(III) center and ${\mathrm{NO}}_{3}^{-}$ , whereby nitrate accelerates oxidative activation, providing the mechanistic basis for designing selective and sustainable C–P bond-forming transformations.

**Scheme 9:**
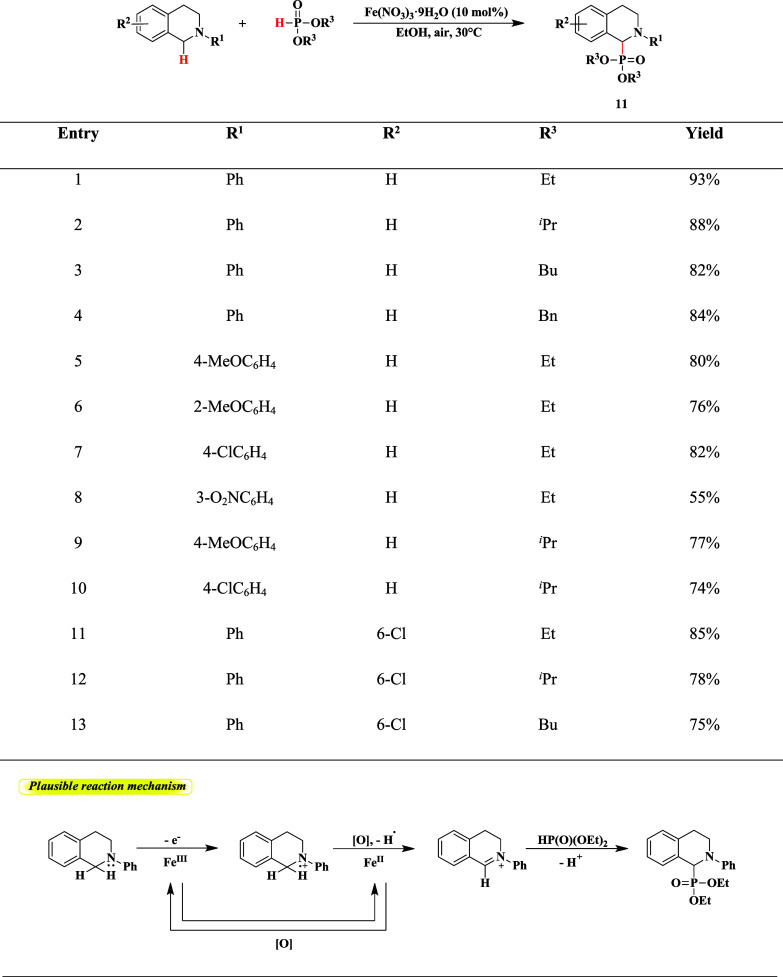
Fe­(NO_3_)_3_·9H_2_O-Catalyzed Aerobic Oxidative Phosphonation of *N*-aryl Tetrahydroisoquinolines

Hu *et al*.^61^ reported a pioneering strategy that integrates alkylation and hydroxylation of C­(*sp* ^*3*^)–H bonds between indolin-2-ones and alkyl-substituted *N-*heteroarenes (Scheme 10). The simultaneous installation of an alkyl substituent and hydroxyl group, with water as the sole by-product, reflects the redox-economical nature of this dual functionalization strategy. The effectiveness of Fe­(OAc)_2_ under ligand-free and base-free conditions suggests that acetate-assisted coordination and controlled radical generation are sufficient to direct selectivity without external additives. The tolerance of sterically encumbered and strongly deactivating substituents, together with ^18^O tracing experiments, implies that molecular oxygen participates directly in the transformation, contributing to functional group installation rather than acting solely as the terminal oxidant.

**Scheme 10:**
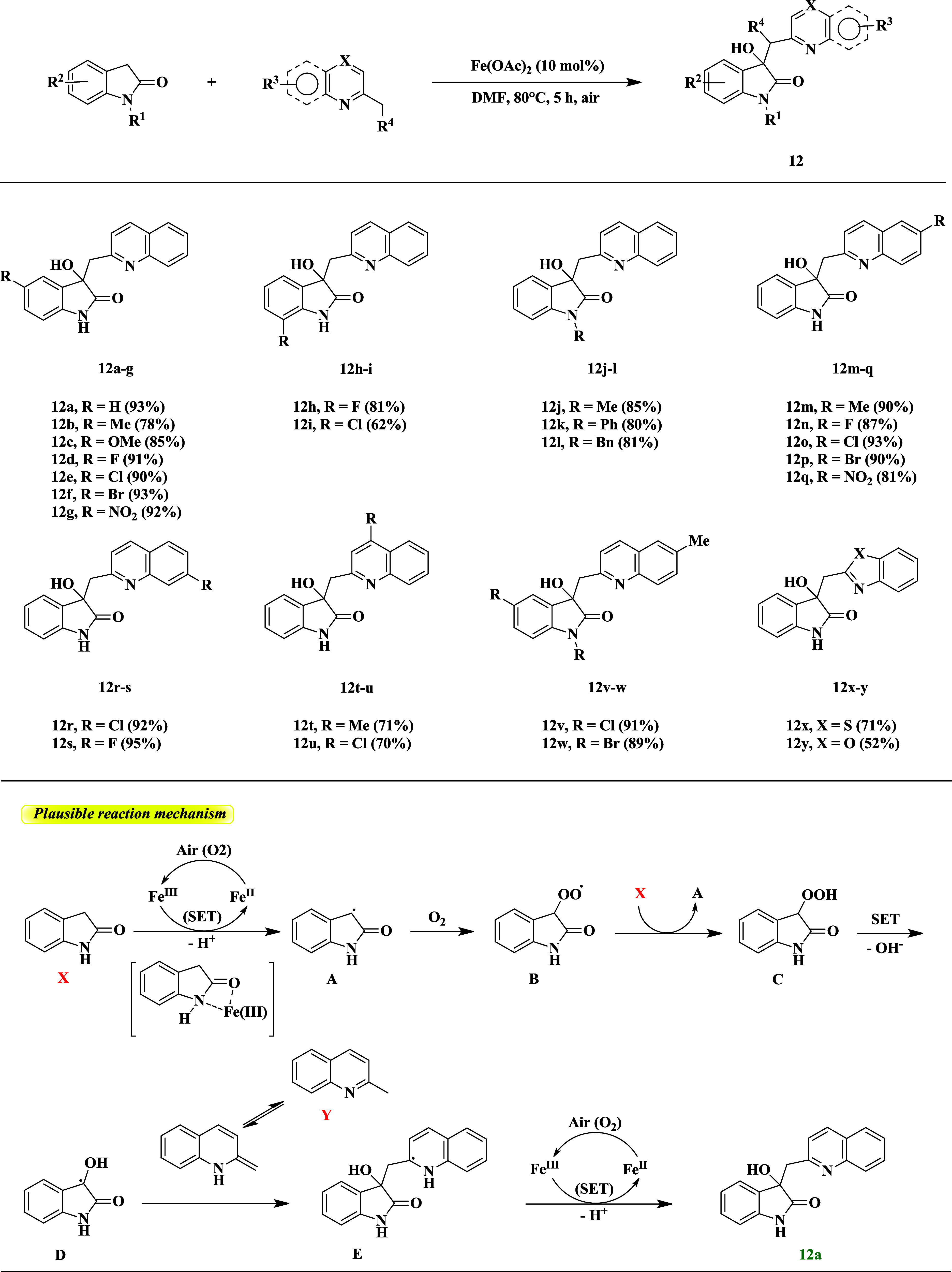
Fe­(OAc)_2_-Catalyzed Direct Oxidative Alkylation and Hydroxylation of Indolin-2-Ones with Alkyl-Substituted *N*-Heteroarenes

### 3.2 Molecular Iron Catalysts with Defined Ligand Environments

An in situ chiral iron complex derived from Fe­(ClO_4_)_2_ and a (1*R*,2*R*)-*N* ^1^,*N* ^2^-di­(quinoline-8-yl)­cyclohexane-1,2-diamine ligand, as detailed by Wu *et al*.,^62^ enabled asymmetric oxidative homocoupling of 2-naphthols, providing direct access to enantioenriched 1,1′-bi-2-naphthols (BINOLs) (Scheme 11). Chirality is imparted by (1*R*,2*R*)-cyclohexane-1,2-diamine within the ligand, which imposes a rigid spatial arrangement around the iron center during the C–C bond formation. The marked solvent dependence, with chlorobenzene outperforming methanol and toluene, suggests that catalyst aggregation and substrate association are highly sensitive to the reaction medium. The significant rate enhancement observed upon the inclusion of 4 Å molecular sieves indicates that trace moisture disrupts productive redox turnover or ligand coordination. Importantly, the perchlorate counterion is weakly coordinating, minimizing competitive ligand binding and allowing the iron center to govern the oxidative pathway. As outlined by the authors, further investigations are underway to design new nitrogen-ligand systems and extend their application across additional transformations.

**Scheme 11:**
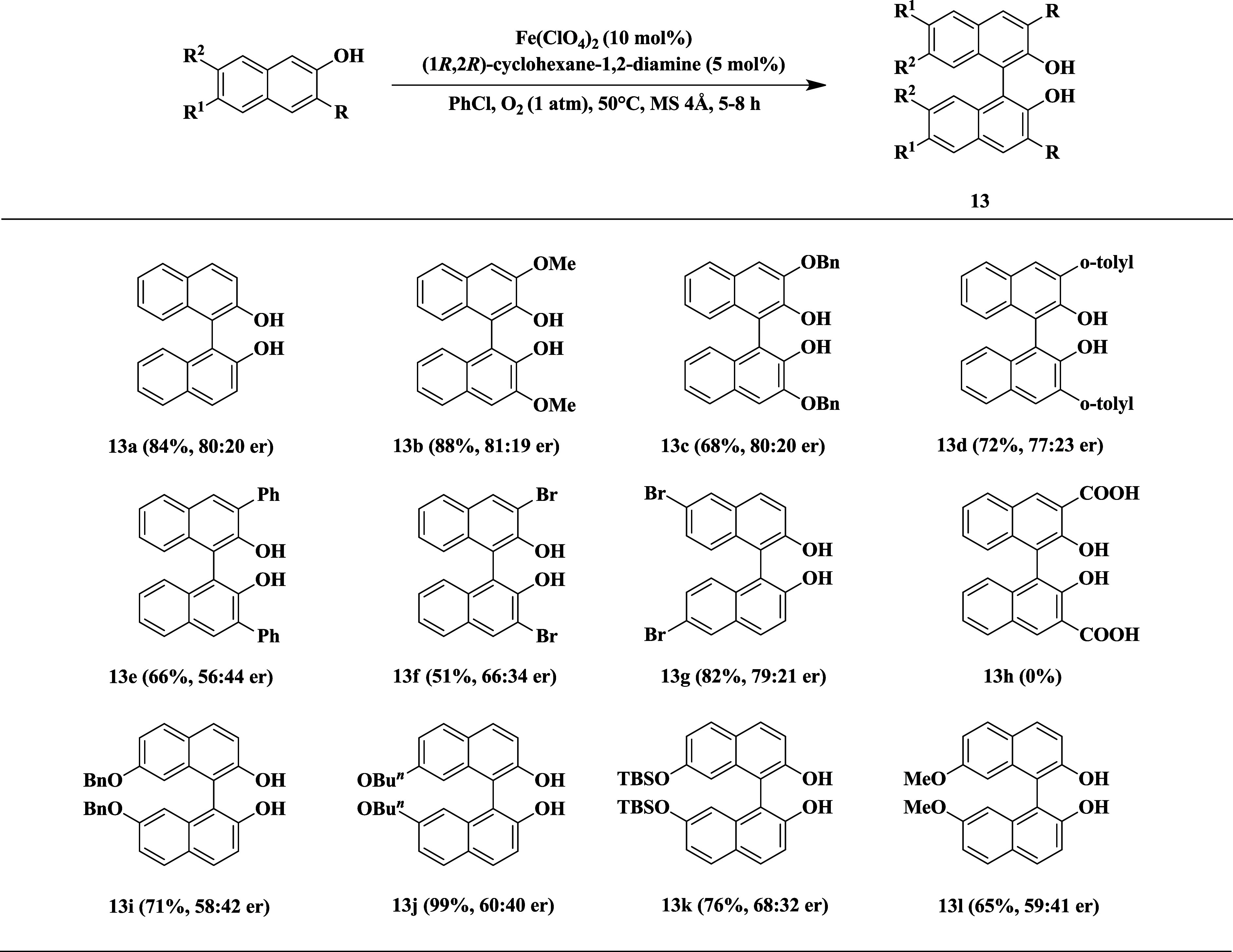
Iron/Bisquinolyldiamine Ligand-Catalyzed Oxidative Coupling of 2-Naphthols

A versatile approach was illustrated by Fritsche *et al*.,^63^ in which strategic modifications to the catalyst structure direct the reaction toward distinct oxidative pathways, affording 2,2′-bis­(arylamino)­biaryls (Scheme 12) and 5,6-dihydrobenzo­[c]­cinnolines (Scheme 13). The consistent failure of porphyrinoid catalysts, including iron tetraphenylporphyrin (FeTPP), indicates a situation wherein reactivity is dictated by the ligand environment, with the metal center playing a secondary role. The observation that weak Brønsted acids such as acetic acid failed to initiate reactivity, but a stronger acid such as *p*-TSA opened up a productive reaction pathway reflects acid-assisted formation of the catalytically active iron species. In this regard, the improved performance of FePcF_16_ appears to stem from electronic modulation of the macrocyclic structure, resulting in superior performance under aerobic conditions. The capacity to guide the construction of C–C and C–N bonds through strategic manipulation of the catalyst and additive parameters reflects a finely balanced interplay between redox activation and substrate binding.

**Scheme 12:**
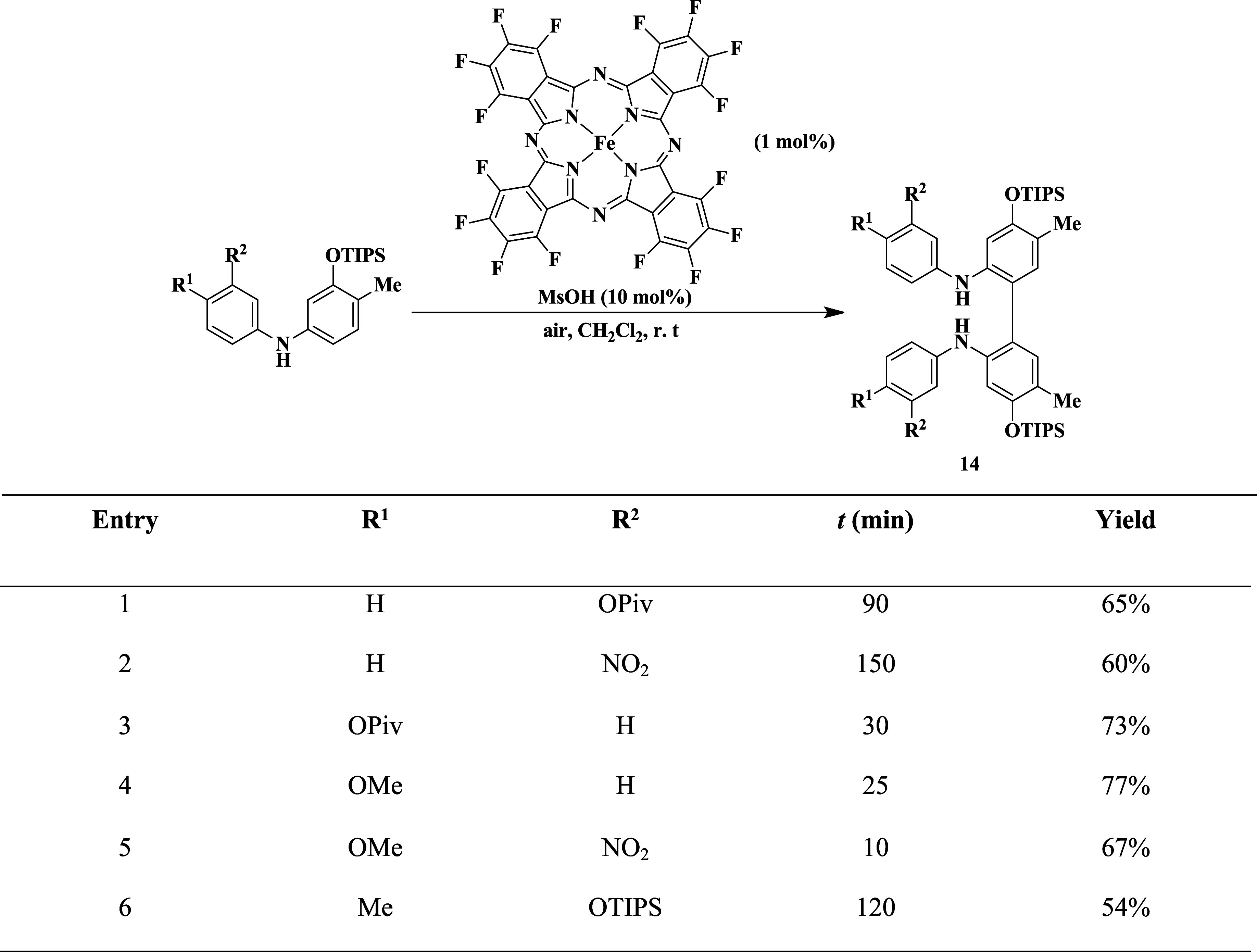
FePcF_16_-Catalyzed Oxidative C–C Coupling of Diarylamines

**Scheme 13:**
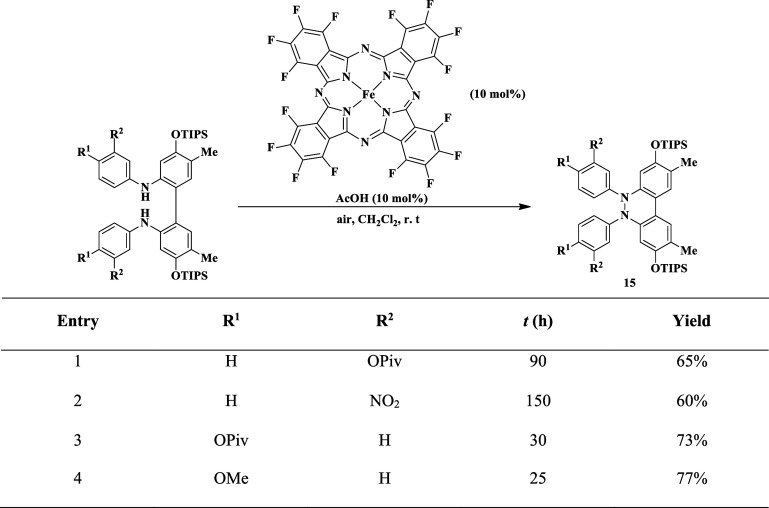
FePcF_16_-Catalyzed Oxidative Cyclization of Biaryl Compounds

A set of Fe phosphasalen complexes was synthesized by Oheix *et al*.^64^ using the phosphasalen ligand Psalen and iron sources (FeCl_3_, Fe­(NO_3_)_3_, Fe­(OTf)_3_). Structural analysis of these complexes identified an iron coordination geometry intermediate between square-based pyramidal and trigonal bipyramidal. Variations in magnetic moments arise from differential axial ligand donor strength, and as a result, Fe­(III) centers retain a high-spin *d* ^5^, while Fe­(II) adopts a high-spin *d* ^6^ state. The coexistence of intraligand transitions of [Fe^II^(Psalen)] and [Fe^III^(Psalen)­(X)] $\left(X=Cl^{-},{\mathrm{NO}}_{3}^{-},{\mathrm{OTf}}^{-}\right)$ at 300 nm and broad LMCT bands between 406 and 497 nm reflects the electronic structure of these complexes, consistent with their moderate ∼50% efficiency of aerobic oxidative coupling of 2-naphthol. The Fe–N and Fe–O bond lengths in the triflate complexes are shorter than those in the chloride complexes, indicating lower donor strength consistent with ligand field effects. Modulation of the axial ligand in iron phosphasalen complexes can regulate redox properties and oxidative coupling efficiency, providing a mechanistic basis for rational catalyst optimization (Scheme 14).

**Scheme 14:**
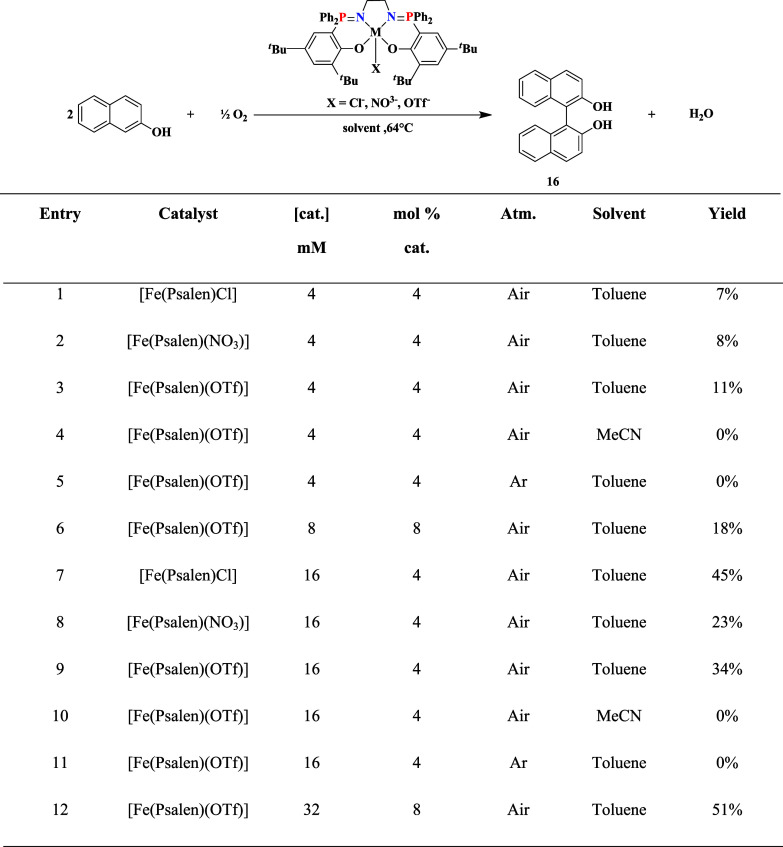
Fe­(Psalen) Complex-Catalyzed Oxidative Coupling of 2-Naphthol

Notable results in oxidative C–C bond construction leading to 1,1′-binaphthyl-2,2′-diamine (BINAM) were revealed by Vershinin *et al*.,^65^ highlighting how *cis*-Fe­[N_4_] complexes impose a distinct reactivity profile. Commercially available aza-crown macrocycles, namely 1,4,7,10-tetraazacyclododecane (cyclen) and 1,4,8,11-tetraazacyclotetradecane (cyclam), furnish structurally well-defined *cis*-Fe­[N_4_] coordination environments, as exemplified by the discrete complexes [Fe­(cyclen)­Cl_2_]Cl and [Fe­(cyclam)­Cl_2_]­Cl, which underpin their effectiveness in controlled oxidative transformation of 2-aminonaphthalenes (Schemes 15, 16). The strong performance observed in 1,1,1,3,3,3-hexafluoropropan-2-ol (HFIP) underscores the role of a strongly organized solvation environment in stabilizing key iron–oxygen intermediates. Notably, *cis*-Fe­[N_4_] complexes including (*S*,*S*′)-[Fe^II^(^dmm^pdp)-(OTf)_2_] and (*S*,*S*′)-[Fe^II^(^TIPS^pdp)-(OTf)_2_], which are established catalysts for asymmetric epoxidation, deliver high conversion yet fail to impart stereochemical bias, pointing to a mechanistic divergence between oxygen transfer and C–C coupling manifolds. A “same-excess” analysis and product-addition experiments collectively demonstrate that the reaction is subject to product inhibition by BINAM rather than catalyst deactivation, while suppression of side reactions in the presence of O_2_ points toward a selective hydroperoxide pathway, thus eliminating the possibility of a radical-based Fenton-type mechanism.

**Scheme 15:**
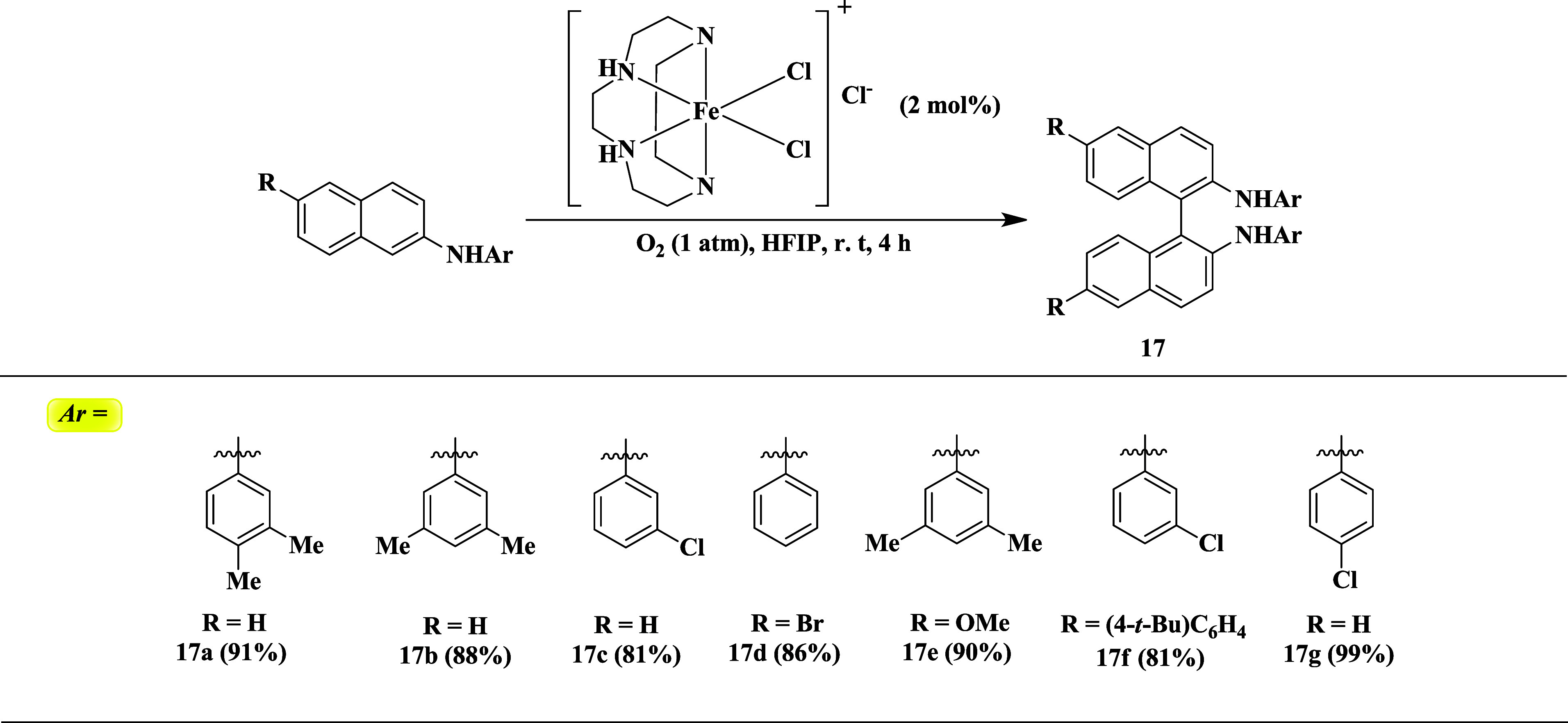
[Fe­(cyclen)­(Cl_2_)]-Catalyzed Oxidative Coupling of *N*-aryl-2-Aminonaphthalenes

**Scheme 16:**
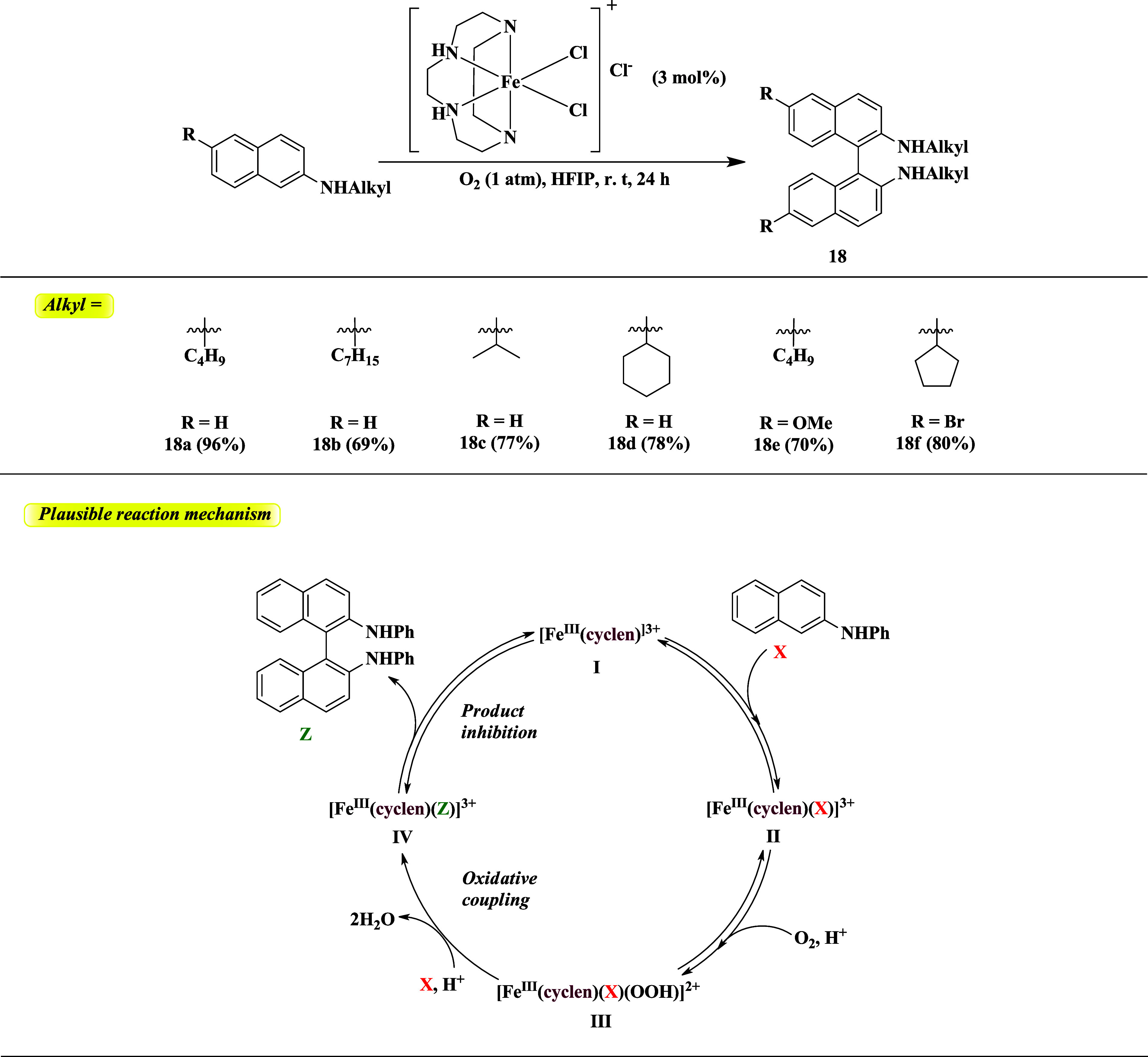
[Fe­(cyclen)­(Cl_2_)]-Catalyzed Oxidative Coupling of *N*-alkyl-2-Aminonaphthalenes

Zhang *et al.* ^66^ established an Fe­(acac)_3_-catalyzed protocol that addresses otherwise conflicting reactivity profiles through systematic reaction design. The combined influence of triethylamine and zinc oxide provides a balanced reaction environment that modulates acidity and enables control over radical interactions, a control system that works even when other metal oxides are used in place of zinc oxide. An important aspect is the system’s ability to reconcile the inherent polarity mismatch between nucleophilic β-keto alkyl radicals and electron-rich enamides, a combination conventionally associated with poor efficiency. Concurrent mitigation of acid-induced enamide self-condensation further preserves productive reaction pathways. The emergence of *Z*-selectivity in this system appears to stem from the inherent geometry of transient intermediates within the catalytic cycle rather than external steric control, demonstrating how finely tuned catalyst-additive cooperation can direct challenging transformations toward stereodefined products under aerobic conditions that are conducive to streamlined synthetic applications (Schemes 17, 18).

**Scheme 17:**
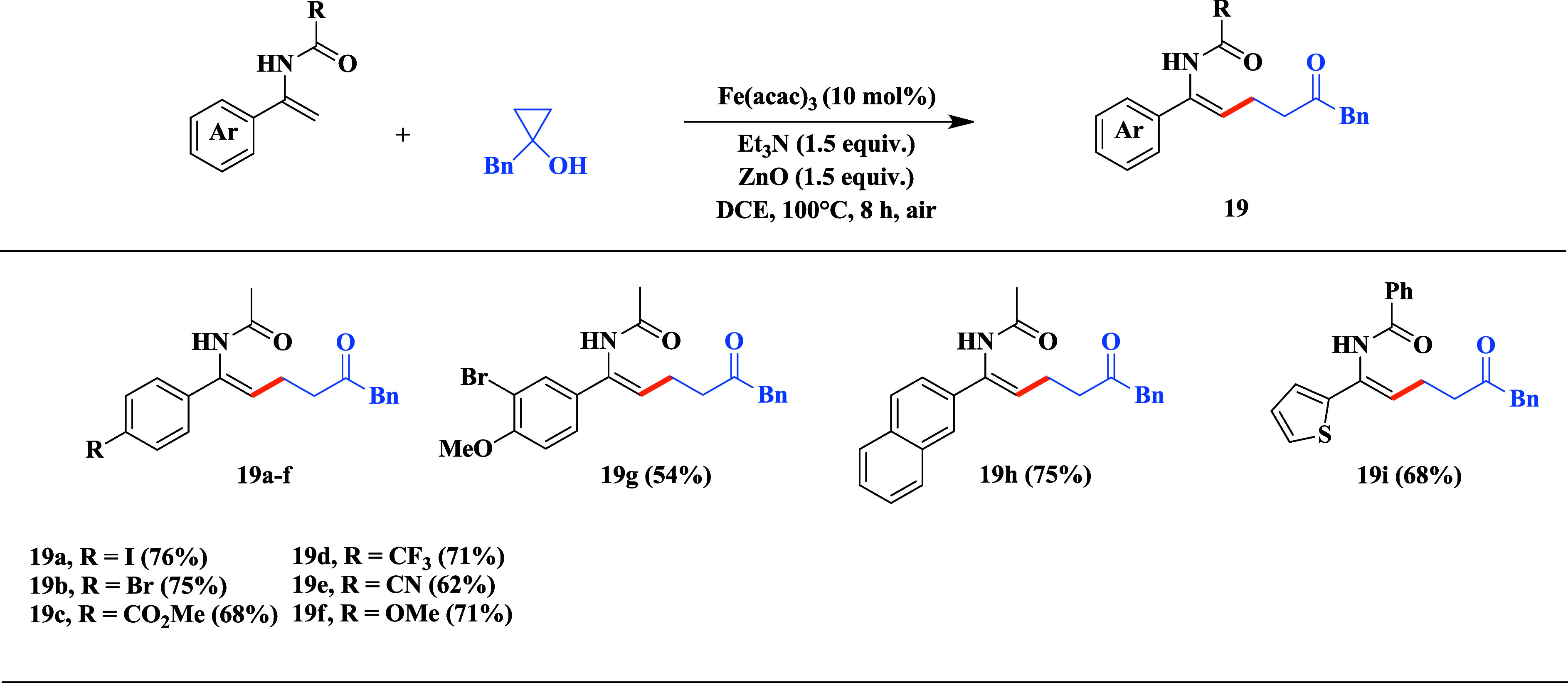
Fe­(acac)_3_-Catalyzed Oxidative Alkylation of *N*-(1-Phenylvinyl)­Acetamide with Cyclopropanols

**Scheme 18:**
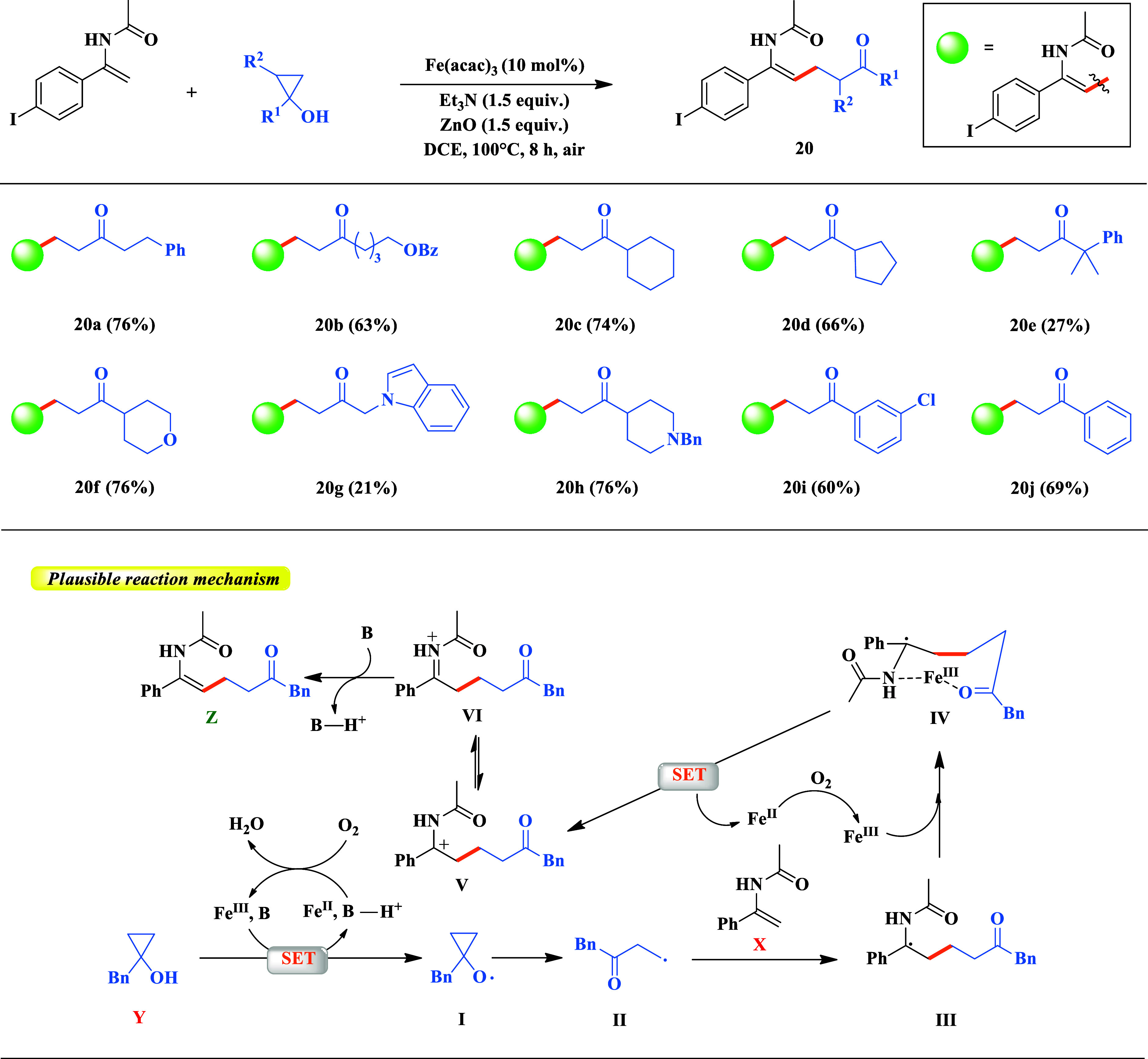
Fe­(acac)_3_-Catalyzed Oxidative Alkylation of *N*-(1-Phenylvinyl)­acetamide with Cyclopropanols

A chiral bipyrrolidine-derived bioinspired nonheme iron complex described by Tkachenko *et al*.^67^ marked the first demonstration of asymmetric aerobic oxidative coupling to deliver enantiomerically enriched BINOLs (up to 47% *ee*) under remarkably low catalyst loadings. The formation of a discrete antiferromagnetically coupled dinuclear mixed-valence complex, assembled *via* coordination of Fe­(ClO_4_)_2_·6H_2_O with the bipyrrolidine-derived aminopyridine ligand in methanol and subsequent acetate incorporation, furnishes a catalytic scaffold structurally suited for direct substrate interaction rather than primary oxidant activation. Kinetic studies reveal a first-order dependence that directly implicates metal–substrate association as the turnover-limiting event. The rate-determining step is the interaction of the iron active sites with the substrates, rather than dissolved oxygen, positioning these complexes as rare functional analogues of oxidase enzymes rather than classical aerobic radical initiators (Scheme 19).

**Scheme 19:**
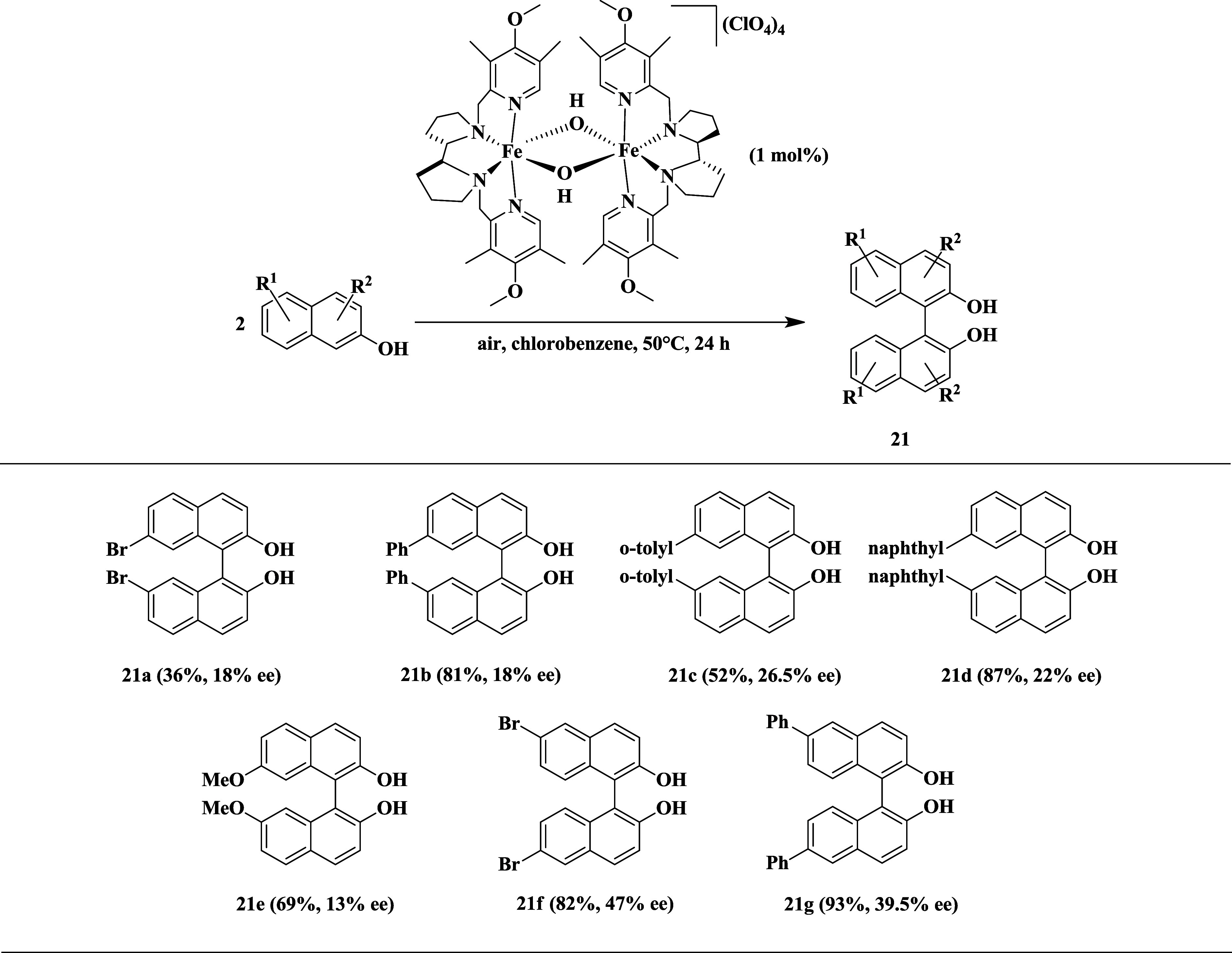
Diferric Aminopyridine Complex-Catalyzed Asymmetric Oxidative Coupling of 2-Naphthols

### 3.3 Heterogeneous Iron-Based Catalysts

Geng and colleagues^68^ synthesized FeO_*x*_/HCMK-3 by immobilizing iron oxide onto mesoporous carbon (HCMK-3) prepared through a hard-template method using SBA-15 and sucrose, thereby imposing structural regularity at the support level. XRD analysis confirms that the long-range mesoscopic order of HCMK-3 is preserved after metal loading, while simultaneously excluding the presence of crystalline iron oxide phases, implying subnanometric dispersion. Direct visualization by STEM, together with energy-dispersive X-ray mapping, corroborates this inference by revealing a uniform spatial distribution of iron without detecting any aggregation. Diffuse reflectance infrared Fourier transform (DRIFT) spectroscopy further clarifies the origin of the stability, indicating strong interactions between the iron species and surface oxygen functionalities that anchor redox-active sites. Yields across the examined substrates ranged from 36.4% to a maximum of 98%, the latter observed for *N*-benzylideneaniline ***(22a)***. Complementary hydrogen temperature-programmed reduction (H_2_-TPR) profiles demonstrate enhanced reducibility, consistent with efficient lattice-oxygen participation during alcohol dehydrogenation and accounting for the catalyst’s strong imine-forming performance (Scheme 20).

**Scheme 20:**
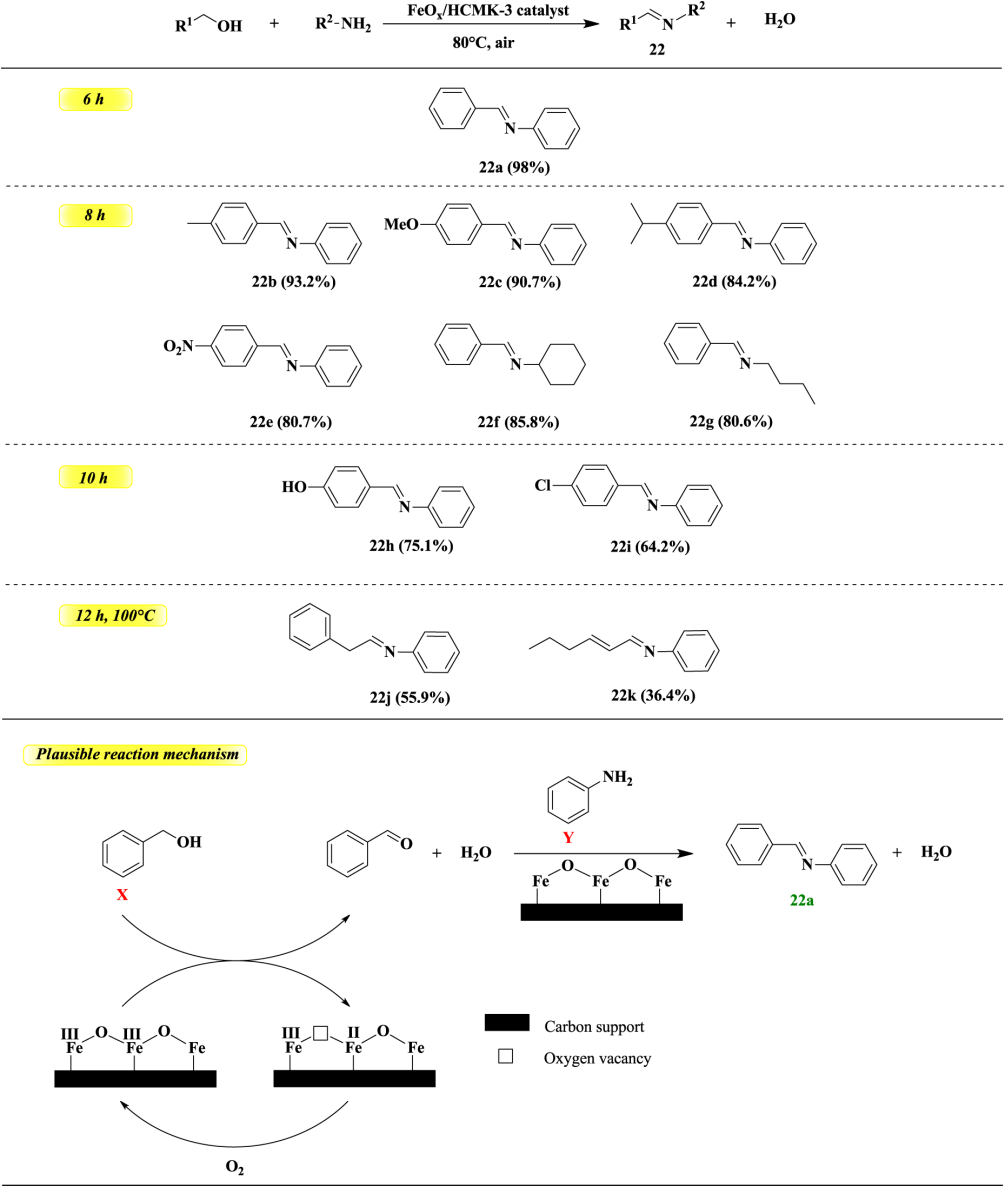
FeO_*x*_/HCMK-3-Catalyzed Oxidative Coupling of Alcohols with Amines

Pyrolytic conversion of FeCl_3_-impregnated wood with urea at 800 °C, as reported by Chen and coworkers,^69^ produced a porous iron single-atom catalyst (Fe-800 SAC) featuring atomically dispersed Fe–N_4_ sites (∼0.8 wt % Fe) incorporated into a defect-rich carbon matrix. Combined spectroscopic analysis, using HAADF-STEM, XANES/EXAFS, and Mössbauer spectroscopy, provides evidence for isolated low-spin Fe^2+^ centers in a square-planar Fe–N_4_ coordination environment embedded within the carbon matrix, while Raman, FT-IR, and solid-state NMR collectively reveal how surface defects and functional groups immobilize these sites against aggregation. The moderate surface area and mesoporous channels inferred from BET analysis ensure efficient mass transport, enabling the Fe–N_4_ motifs to outperform a wide array of competing SACs (Fe-600, Fe-700, Fe-900, Co-800, Ni-800, and Cu-800) examined in three-component quinoline synthesis. Corroborated by spectroscopic signatures and DFT analysis, the catalytic advantage arises from cooperative O_2_ and carbonyl activation at Fe–N_4_ single-atom sites, consistent with the observed durability over eight consecutive cycles without detectable leaching or restructuring (Scheme 21).

**Scheme 21:**
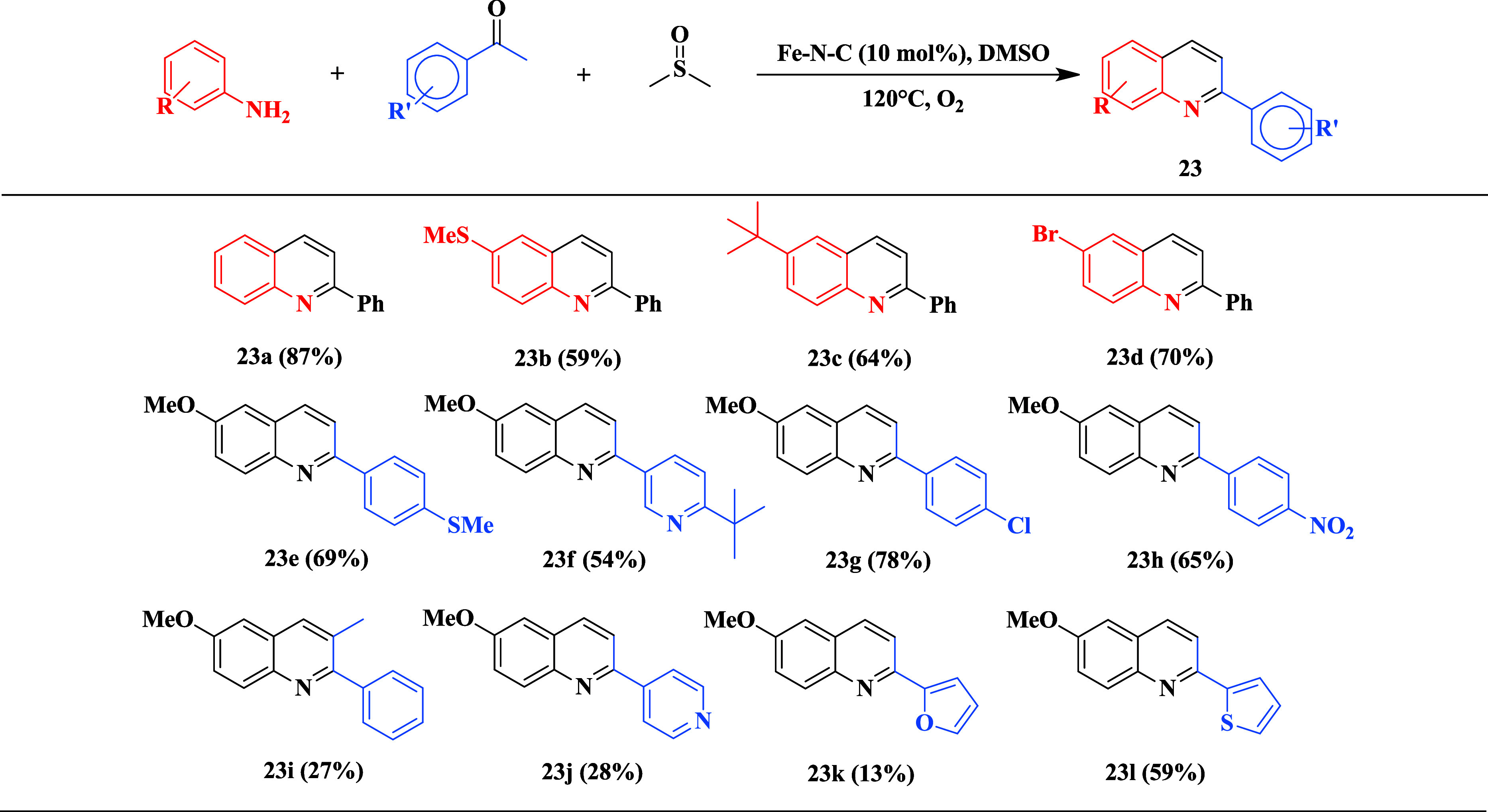
Three-Component Iron Single Atom-Catalyzed Synthesis of Quinolines

Phase-pure ternary CoNiFe layered double hydroxide (CoNiFe-LDH), an anionic clay composed of mixed Co^2+^/Ni^2+^/Fe^3+^ hydroxide layers, was synthesized by Xia and colleagues,^70^ with structural integrity verified by XRD analysis. Within the ordered hydrotalcite framework, the close spatial integration of the three redox-active metal centers enables a cooperative regulation of oxidation and C–P bond-forming steps during the oxidative phosphorylation of α-amino C–H bonds (Scheme 22). Systematic screening of mono- and bi-metallic analogues (CoAl, CoFe, CoGa, MgFe, NiAl, and NiFe) revealed that cobalt and iron jointly drive iminium formation, while nickel predominantly modulates selectivity, collectively establishing that no single metal center alone is responsible for the observed catalytic efficiency. This synergistic interplay accounts for the superior efficiency of CoNiFe-LDH relative to mono- or bi-metallic counterparts and illustrates how lattice-level metal cooperation can be leveraged to achieve selective C–P bond formation in a recyclable, additive-free heterogeneous system.

**Scheme 22:**
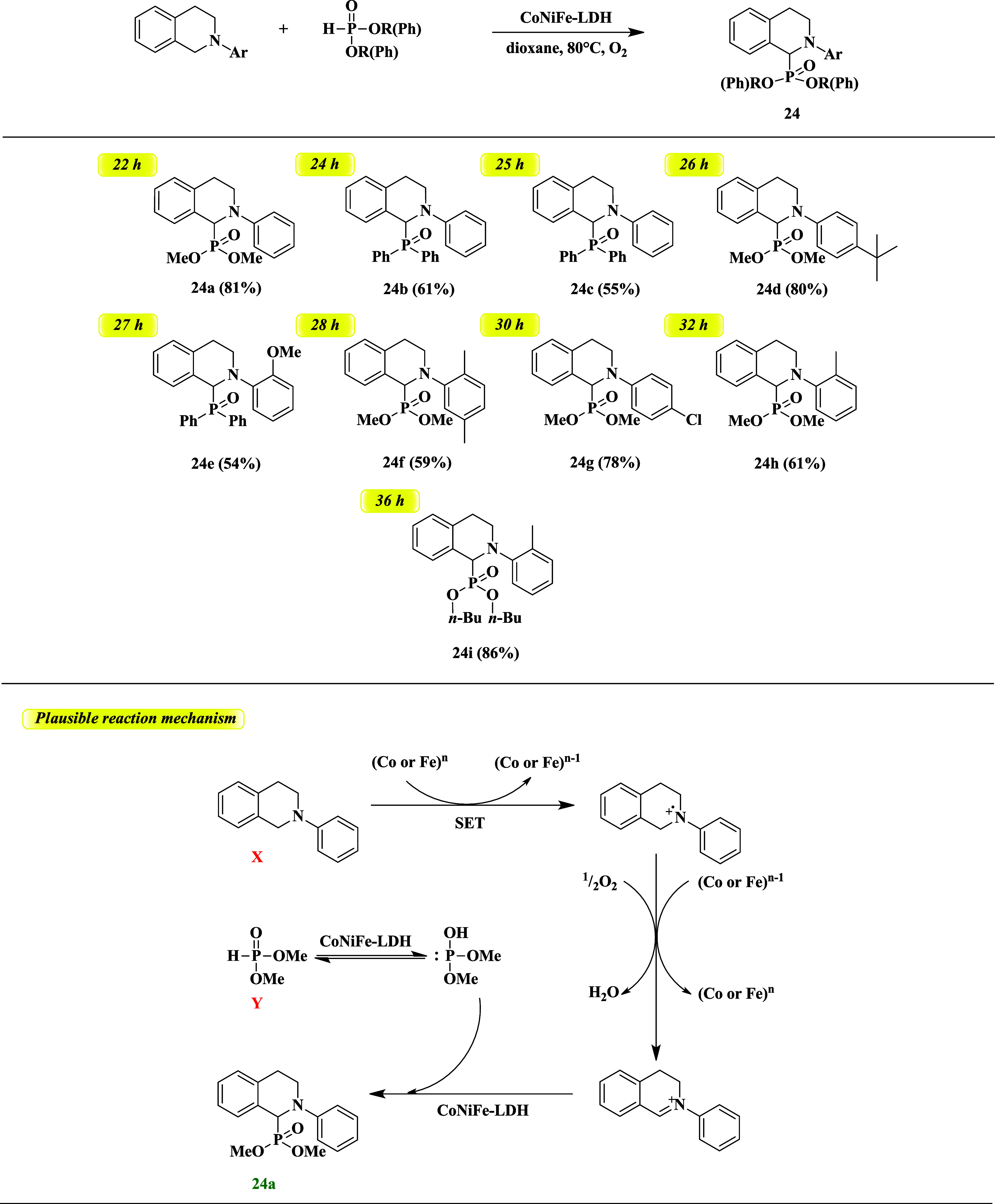
CoNiFe Hydrotalcite-Catalyzed Oxidative Phosphonation of α-Amino C–H Bonds

As reported by Shaker and Elhamifar,^71^ the use of a magnetically recoverable Fe_3_O_4_@methylene-bridged mesoporous organosilica-ionic liquid/Pd (Fe_3_O_4_@MePMO-IL/Pd) composite demonstrates the influence of framework design and redox accessibility on catalytic performance, enabling the formation of biphenols and BINOLs. FT-IR analysis clarifies that iron oxide incorporation occurs without disrupting the hybrid siliceous backbone, while wide-angle PXRD establishes the presence of crystalline iron oxide domains and low-angle PXRD patterns confirm preserved mesoscopic order. Reactivity trends further imply that substrate-dependent oxidation potentials dictate coupling selectivity, with naphthol systems progressing through well-defined radical manifolds, whereas phenolic analogues diverge toward competing resonance-stabilized pathways. Notably, this system represents an informative case where the Fe_3_O_4_ magnetic core assumes the structural, separation, and support role rather than a primary catalytic one, contributing to magnetic recoverability and enhanced substrate accessibility through its methylene-bridged mesoporous framework, with the oxidative coupling being unambiguously assigned to the palladium species. This is conclusively established by three decisive control experiments in which Fe_3_O_4_@MePMO-IL without Pd, Fe_3_O_4_@MePMO without Pd or IL, and Fe_3_O_4_@SiO_2_ without Pd each afforded no product, confirming that the absence of palladium entirely abolishes reactivity. Hot filtration at ∼50% conversion halted any further reactivity upon magnetic separation of the solid, verifying a truly heterogeneous and readily recoverable catalyst, while overall, the results highlight how embedding iron oxide within a mesoporous hybrid scaffold creates an efficient oxidative coupling platform through high surface accessibility and durable redox-active centers (Scheme 23).

**Scheme 23:**
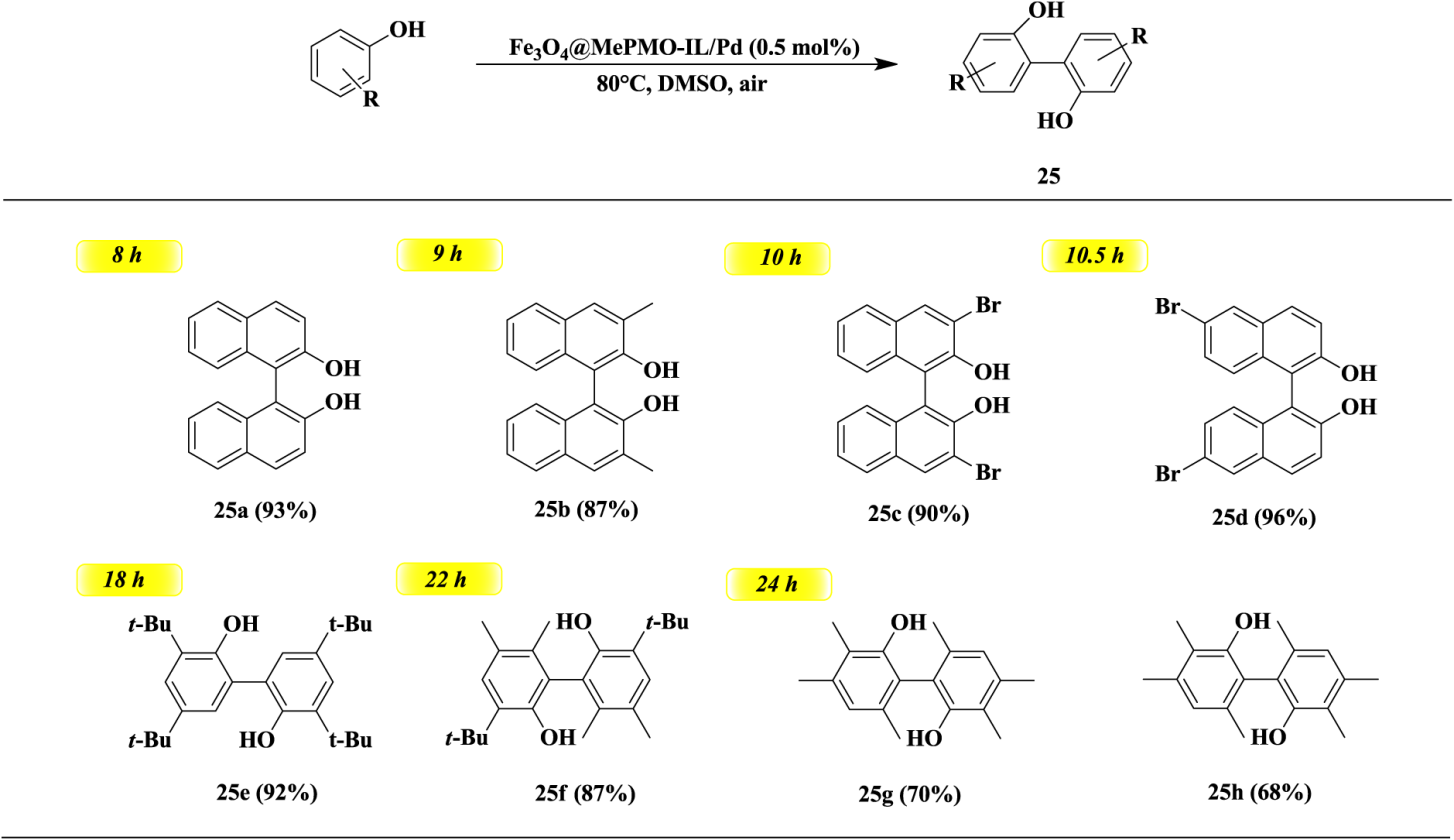
Fe_3_O_4_@MePMO-IL/Pd-Catalyzed Oxidative Coupling of Phenols and Naphthols

Nguyen *et al.* ^72^ introduced an efficient and magnetically recoverable spinel CuFe_2_O_4_ that illustrates how the bimetallic spinel framework governs aerobic C–C bond construction from chalcones and 2-aminopyridines/pyrimidines. The reliance on multinuclear ^1^H, ^13^C, and ^19^F NMR techniques confirms the structural identity of the products. Crucially, the spinel structure itself is the active entity, as substitution with discrete CuO or Fe_2_O_3_ nanoparticles of equivalent metal content or with mononuclear copper salts (CuI, CuBr, Cu­(OAc)_2_) and iron salts (FeCl_2_, FeCl_3_) used individually afforded only trace or inferior yields, establishing that the Cu–Fe oxide framework is synergistic and structurally essential, with neither metal alone capable of reproducing the reactivity of the intact spinel. Solvent effects further suggest that excessive coordination, as in DMF or DMSO, attenuates catalytic turnover, whereas 1,4-dioxane preserves active site accessibility. Mechanistically, iodine-enabled α-iodoketone generation directs reactivity toward cyclization, leaving CuFe_2_O_4_-mediated aerobic C–C oxidation as the kinetic bottleneck, consistent with its retained activity over 5 recycling cycles (Scheme 24).

**Scheme 24:**
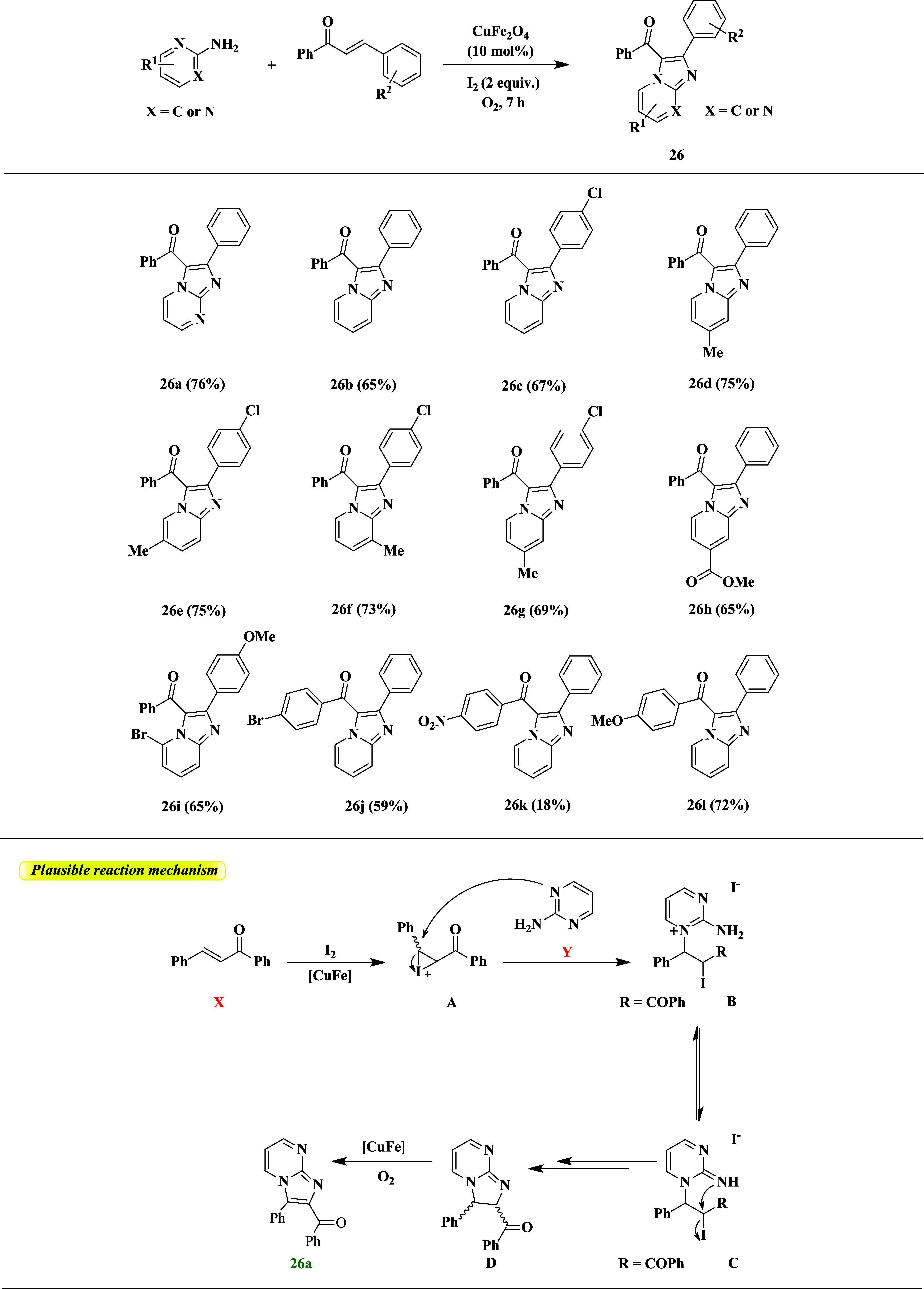
CuFe_2_O_4_-Catalyzed Oxidative Coupling of 2-Aminopyridines/Pyrimidines with Chalcones

Baruah *et al.* ^73^ developed a facile method for the synthesis of an iron oxide-halloysite nanotube (Fe_2_O_3_-HNT) hybrid catalyst, where Fe_2_O_3_ nanoparticles were immobilized within the lumen of naturally occurring halloysite clay, and the authors demonstrated their application in visible-light-driven oxidative coupling of 2-naphthols. The structural analyses, including FT-IR and XRD, revealed that the halloysite framework and crystallinity were preserved upon Fe_2_O_3_ incorporation, while TEM and FESEM confirmed uniform nanoparticle dispersion confined within the nanotube channels. XPS established iron predominantly in the Fe^3+^ state, and UV-Vis diffuse reflectance spectroscopy with Tauc plot analysis indicated a band gap of ∼2.4 eV, suitable for visible-light activation. Significant quenching in photoluminescence spectra suggested enhanced charge separation, which accounts for the high photocatalytic efficiency. The ability to tune oxidative coupling yields by modulating O_2_ flow rates further highlights the interdependence between oxygen availability and surface-bound Fe­(III)-superoxide generation. Collectively, these observations underscore the advantage of spatially confined Fe^3+^ sites in halloysite for sustainable, low-cost photocatalysis (Scheme 25).

**Scheme 25:**
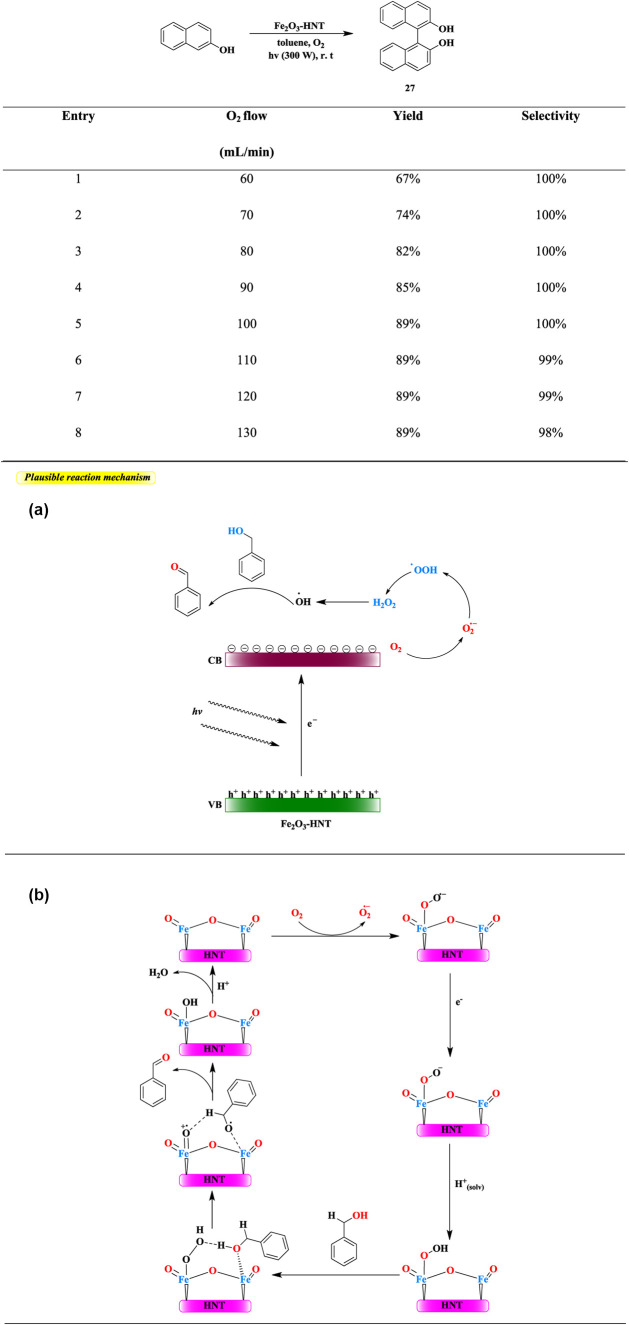
Fe_2_O_3_-HNT-Catalyzed Visible-Light-Assisted Oxidative C–C Coupling of 2-Naphthol; (a) Free Radical Mechanism; (b) Conversion through Initial Substrate Adsorption on the Catalyst Surface

### 3.4 Cooperative and Multicomponent Iron Catalytic Systems

Dey *et al.* ^74^ presented a direct, atom-efficient, and concise synthetic strategy for constructing angularly fused furan derivatives through the coupling of arylacetylenes with enolic substrates. In this system, the yield of 2-phenyl-4*H*-furo­[3,2-*c*]­chromen-4-one ***(28a)*** increases to 80% once ZnI_2_ is introduced, implying that a Lewis acid is involved in the stabilization of radical intermediates rather than serving as a second redox-active center. Iron remains conclusively the species driving the radical oxidative mechanism, as established by radical scavenger-induced suppression of product formation and the complete absence of reactivity when ZnI_2_ is used alone without FeCl_3_. The reaction therefore follows a defined addition and ring closure sequence in which iron and zinc play distinct and complementary roles, with the iron catalyst alternating between oxidation states to sustain the turnover process (Scheme 26).

**Scheme 26:**
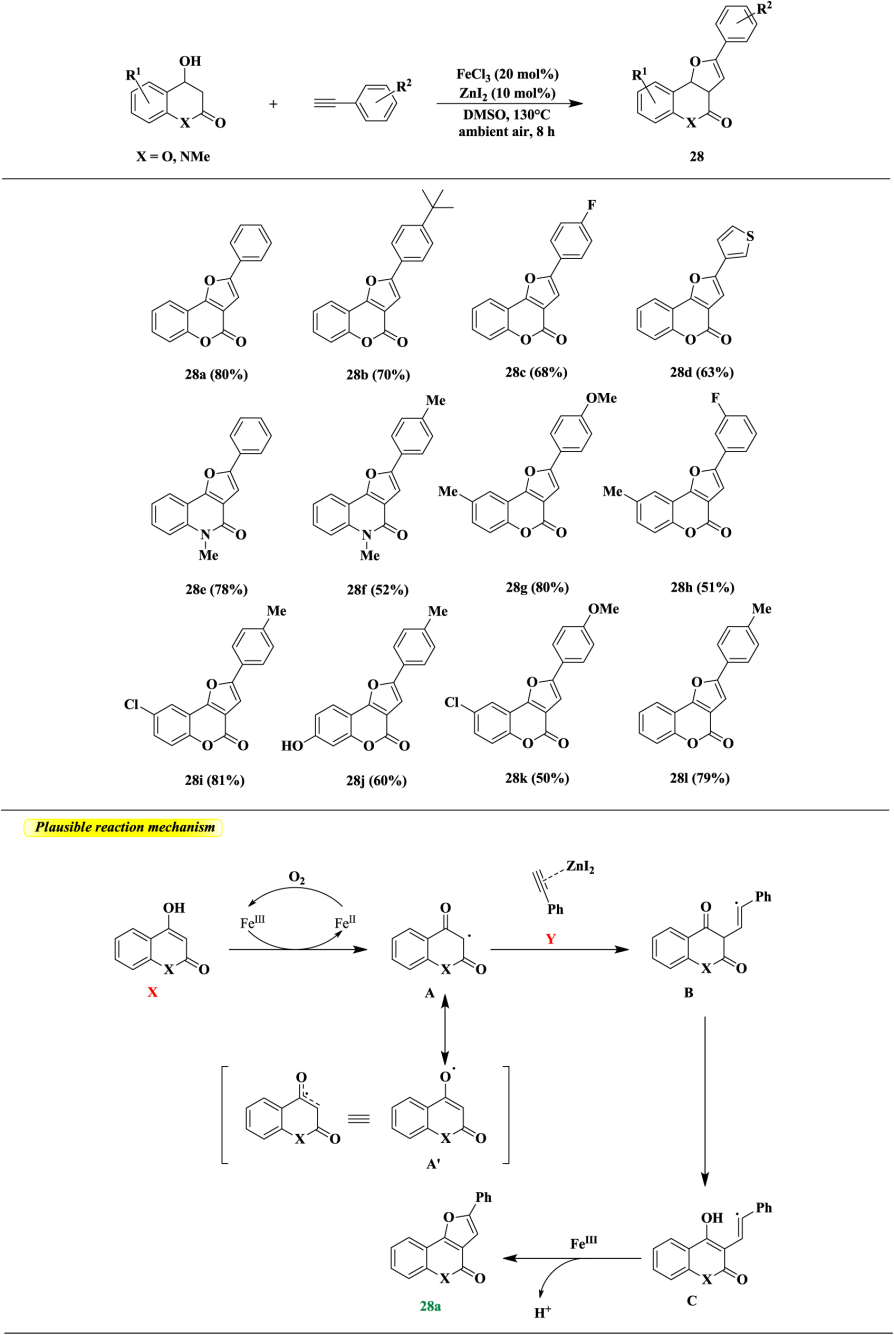
FeCl_3_/ZnI_2_-Catalyzed Regioselective Synthesis of Angularly Fused Furans

A study by Zhang and colleagues^75^ on the biomimetic catalytic oxidative coupling of thiols using a thiolate-bridged iron–ruthenium complex indicates that structural flexibility is central to sustained turnover. Spectroscopic characterization supported by ^1^H and ^13^C NMR, ESI-HRMS, IR, elemental analysis, and single-crystal X-ray diffraction reveals an unusual coordination mode in which the thioether sulfur atom migrates between metal centers, consistent with the conformational flexibility of the tpdt ligand, implying adaptive metal–sulfur communication during redox cycling. Two key catalytic intermediates, the phenylthiolate-coordinated complex and the terminal hydride complex, were directly characterized by X-ray crystallography, establishing the clear division of labor in which iron serves as the primary oxygen-activating center while ruthenium mediates thiol coordination and hydride management, a cooperation further confirmed by the substantially lower activity of [CpFe­(η^3^-tpdt)] and [CpRu­(η^3^-tpdt)] used individually and the near-complete inactivity of the iron–cobalt analogue, demonstrating that the Fe–Ru combination is uniquely productive. This cooperative Fe–Ru synergy stands in contrast to the initially explored tpdt-bridged diiron complex, [Cp*Fe­(μ-tpdt)­FeCp*]-[PF_6_], which afforded 100% yield for the oxidative coupling of thiophenol to diphenyl sulfide ***(31a)*** in THF. However, it is worth noting that this diiron complex decomposed into unidentified insoluble species after the reaction, whereas 2­[PF_6_] also served as an excellent catalyst (100% yield), maintaining its binuclear structure post-reaction. Motivated by environmental and sustainability considerations, the authors developed a greener reaction platform in which the oxidative coupling of thiophenol catalyzed by 2­[PF_6_] proceeded in water using oxygen as the oxidant, albeit with a longer reaction time of 3 h and a slight reduction in yield to 90%, reflecting a compromise between stability and reactivity (Scheme 27).

**Scheme 27:**
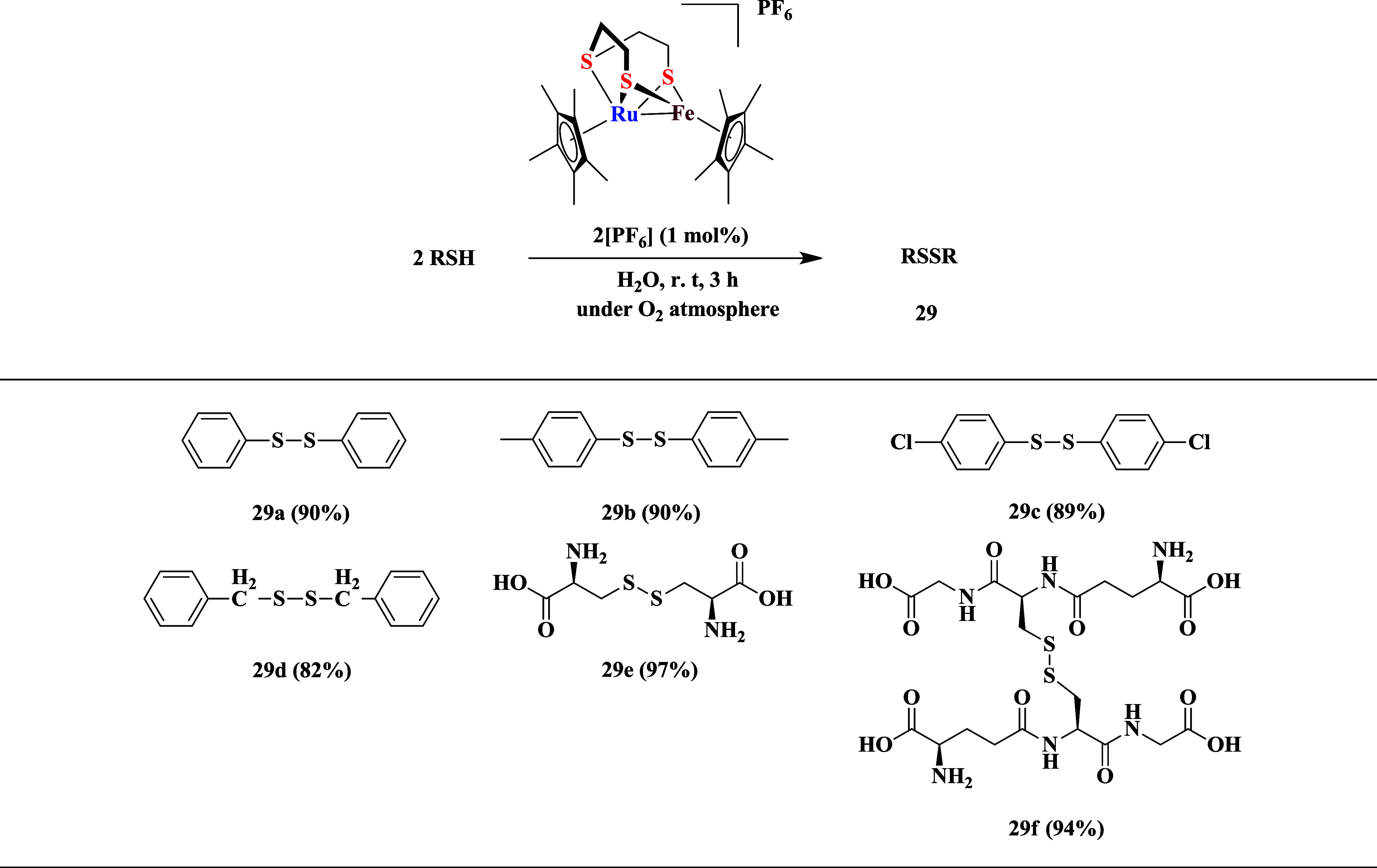
Tpdt-Bridged Iron–Ruthenium Complex-Catalyzed Oxidative Coupling of Thiols

A two-component catalytic system was developed by Zhang *et al.* ^76^ to enable the direct aerobic oxidative coupling of methyl-substituted *N*-heteroazarenes with alcohols (Schemes 28, 29). Rather than operating as independent processes, the oxidation and condensation steps are kinetically interdependent, with their balance representing a key challenge. Under these conditions, the Fe­(NO_3_)_3_·9H_2_O/TEMPO system enables controlled oxidation and maintains selectivity toward *E*-substituted olefins. The operational role of *t-*BuOK extends beyond simple deprotonation, as the base also promotes the formation of enamine species from the heteroarene precursor. Concurrently, the inclusion of 18-crown-6 enhances the solubility of *t-*BuOK in THF, indirectly regulating reaction progression through improved phase homogeneity and catalytic efficiency.

**Scheme 28:**
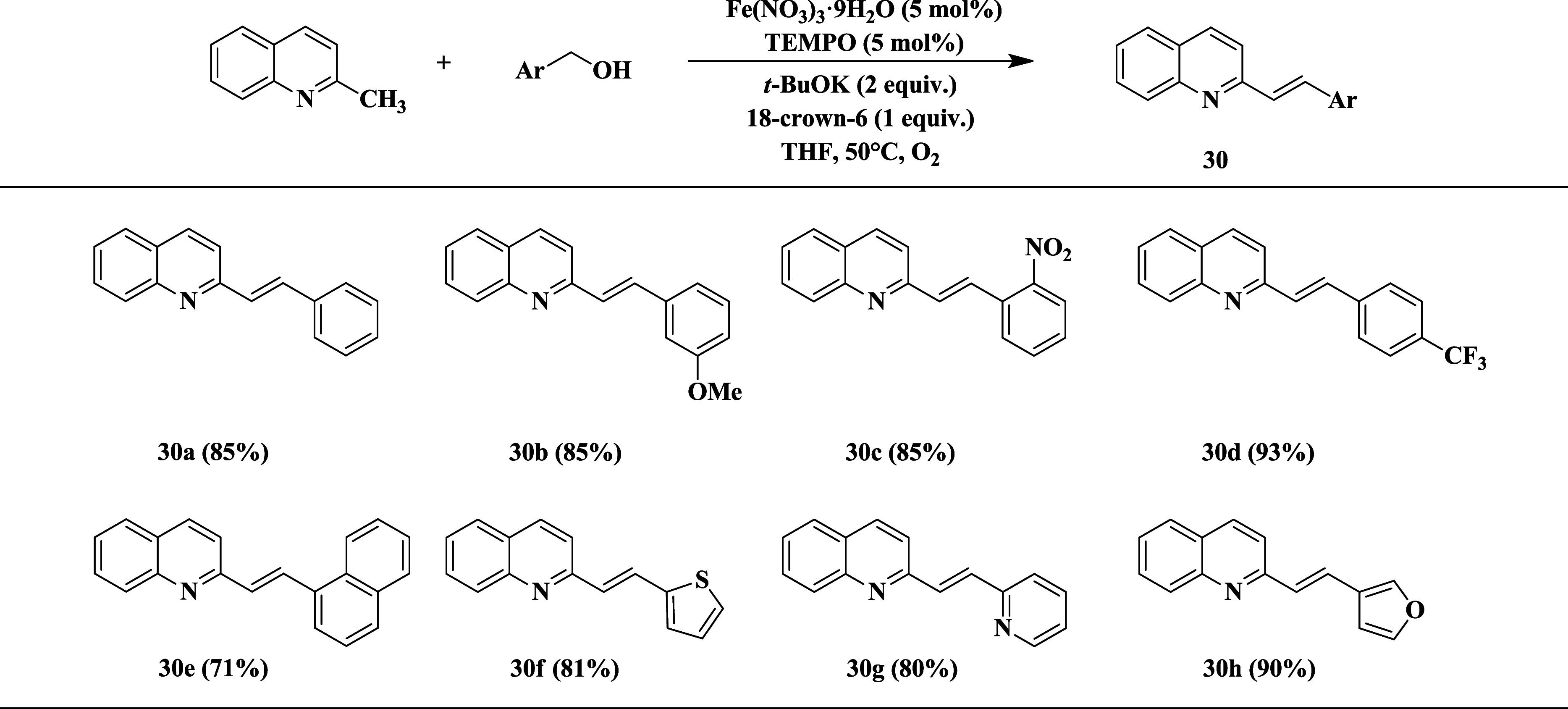
Iron/TEMPO-Catalyzed Direct Aerobic Oxidative Coupling of 2-Methlquinline with Alcohols

**Scheme 29:**
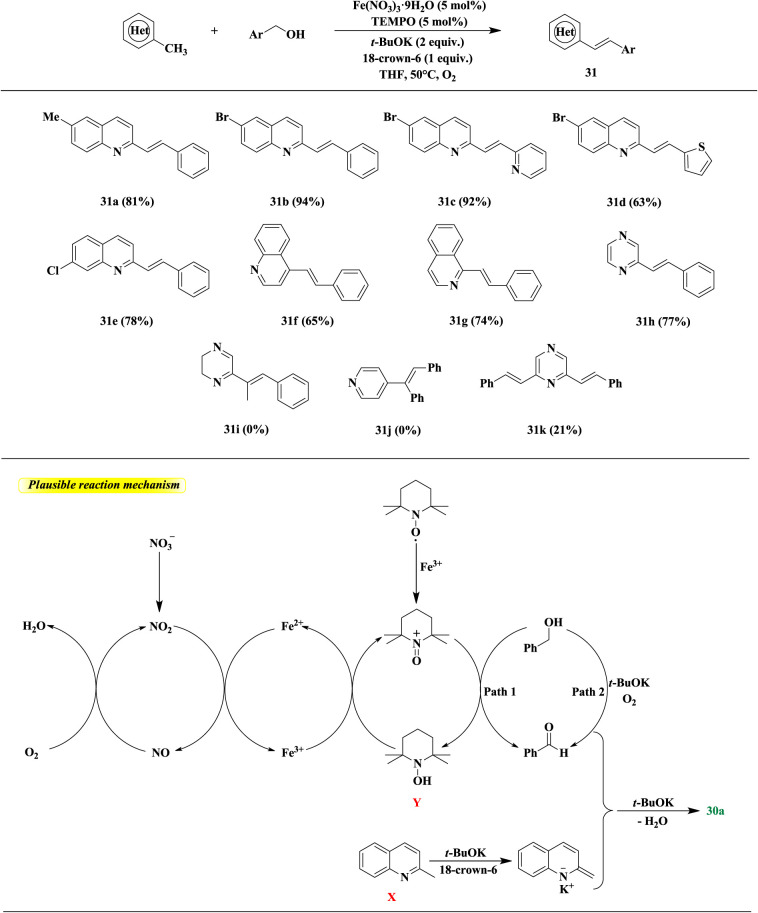
Iron/TEMPO-Catalyzed Direct Aerobic Oxidative Coupling of *N*-Heteroazaarenes with Alcohols

Kumar *et al*.^77^ successfully synthesized the Fe­(bpy)_3_/npg-C_3_N_4_ photocatalyst, which involved the immobilization of the iron­(II) bipyridine complex onto a nanoporous graphitic carbon nitride framework, forming a heterojunction between the npg-C_3_N_4_ π-electron sheet and the metal complex, and creating a synergistic interaction that enhances the photocatalytic activity for the synthesis of imines. SEM first outlines surface topology evolution, while TEM clarifies nanoscale dispersion behavior and confirms intimate interfacial contact. FT-IR then verifies coordination environments through ligand vibrational modes, followed by XRD, which demonstrates preservation of crystalline order after integration. UV-Vis spectroscopy reveals the Fe­(II) tris­(bipyridine) MLCT transition together with a pronounced red shift and broadened absorption profile, consistent with efficient visible-light activation. The electronic coupling between the inorganic support and molecular iron center is proposed to sustain catalytic turnover, with operational consistency across six consecutive runs reinforcing structural integrity and high imine yields under photochemical oxidative conditions (Scheme 30).

**Scheme 30:**
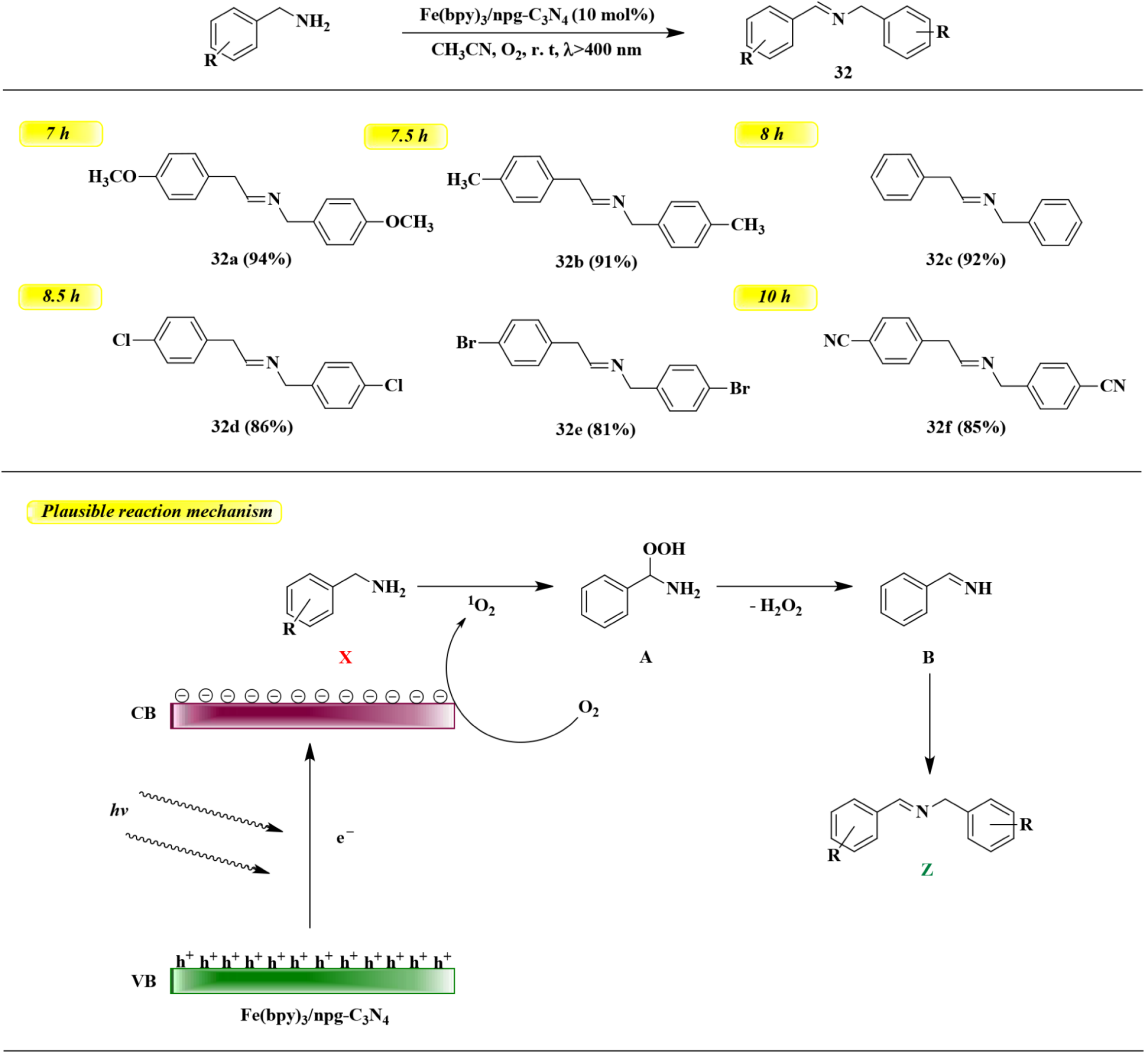
Fe­(bpy)_3_/npg-C_3_N_4_-Catalyzed Visible-Light-Assisted Oxidative Coupling of Benzylamines

An environmentally benign iron-catalyzed aerobic oxidative coupling strategy was reported by Zhang and coworkers,^78^ utilizing an Anderson-type polyoxometalate (POM) inorganic ligand-supported catalyst, (NH_4_)_3_[FeMo_6_O_18_(OH)_6_], representative of the {MMo_6_O_18_(OH)_6_} (M = transition metals) structural motif. Within this framework, iron serves as the sole redox-active center, a role established through three converging lines of evidence: neither Fe_2_(SO_4_)_3_ nor (NH_4_)_6_Mo_7_O_24_·4H_2_O alone afforded any product even after 48 h, establishing that molybdate is not an independent catalytic center but rather an electron-withdrawing inorganic scaffold that enhances the electrophilicity of the iron core. Consistent with this, hybrid derivatives featuring varying peripheral organic substituents on the same {Fe^III^Mo_6_} core all delivered similarly high yields, confirming that the organic ligand environment exerts negligible influence on reactivity and that catalytic activity resides exclusively within the inorganic Fe–Mo oxide framework. Operational compatibility across both self-coupling of primary amines and cross-coupling between benzyl alcohol and amines indicates mechanistic convergence at a common oxidation platform. Formation of Fe­(V)-oxo, which serves as the key reactive intermediate, accounts for the selective substrate activation, while GC–MS monitoring provides direct support for an aldehyde-mediated oxidative coupling pathway. Catalyst recovery by simple hot filtration, with infrared analysis confirming the preservation of the Fe–Mo oxide framework after catalysis, demonstrates structural stability under turnover conditions (Schemes 31, 32).

**Scheme 31:**
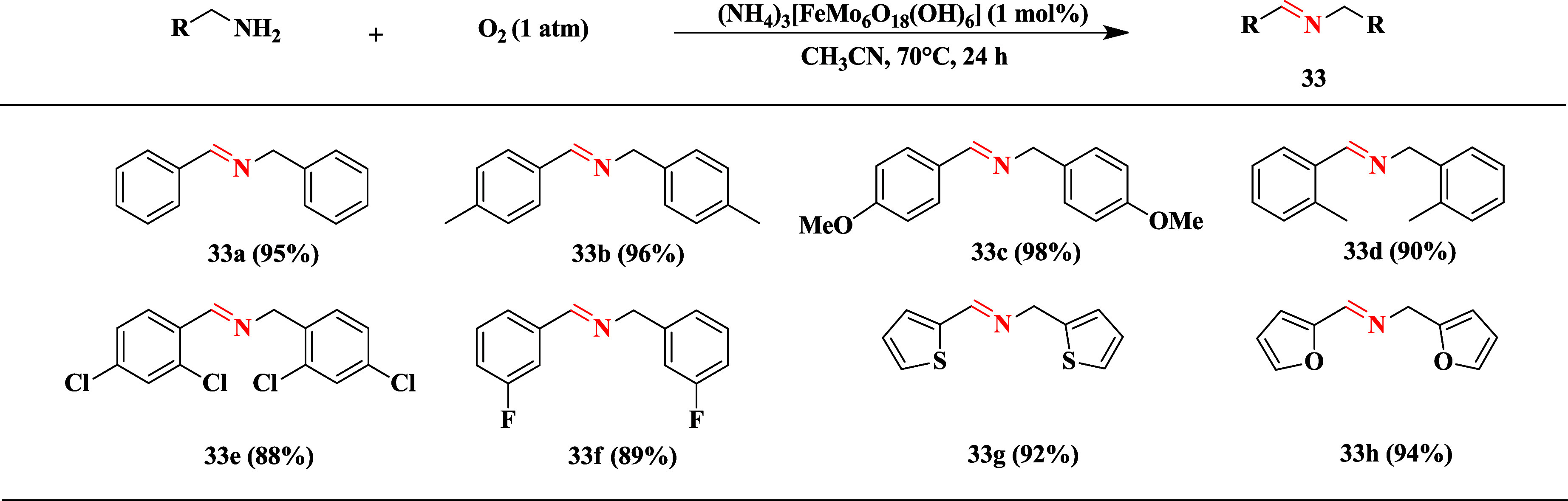
Inorganic-Ligand Supported Iron-Catalyzed Self-Coupling of Amines

**Scheme 32:**
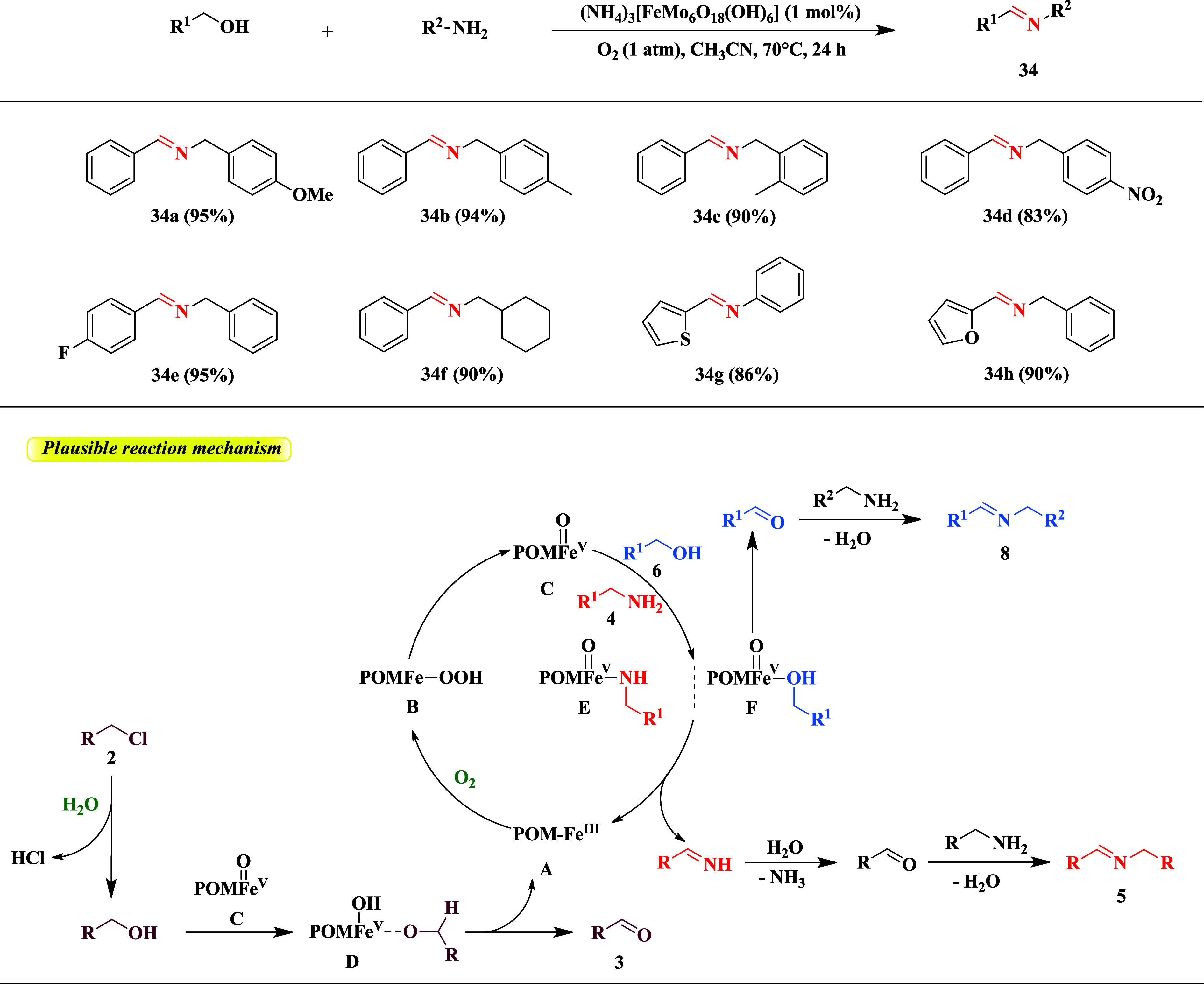
Inorganic-Ligand Supported Iron-Catalyzed Cross-Coupling of Amines with Alcohols

## 4 Key Limitations, Challenges, and Potential Solutions

A recurring limitation across iron-catalyzed oxidative coupling methods is the reduced reactivity of electron-deficient substrates. Strongly electron-withdrawing substituents consistently delivered diminished yields across homogeneous iron salt catalysts, iron phthalocyanine complexes, and heterogeneous iron oxide-supported systems. This electronic sensitivity reflects the dependence of radical or SET initiation on substrate oxidizability, meaning that partners with reduced electron density resist the initial oxidation step. Aliphatic and sterically hindered substrates face related difficulties as their lower radical stability and restricted access to the iron center further limit productive turnover. Addressing this limitation will likely require the rational design of iron complexes with electronically tuned ligand frameworks capable of lowering the activation barrier for electron-deficient coupling partners without compromising the mild and sustainable character of these systems.

The electronic constraints on substrate scope are intimately connected to a broader mechanistic vulnerability, namely, that the radical nature of iron-catalyzed oxidative couplings, while mechanistically enabling, introduces inherent selectivity challenges. Radical scavenger experiments across the surveyed catalyst systems consistently confirmed free radical involvement while simultaneously revealing the ease with which product pathways are diverted. Competing processes identified across the review literature include homocoupling, over-oxidation to carboxylate or amide byproducts, uncontrolled intramolecular cyclization, and poor regioselectivity in phenoxy radical recombination. Suppressing these pathways will require more precise radical lifetime control, achievable through photoredox cocatalysis, bulkier ligand frameworks that sterically bias radical recombination, or carefully tuned oxygen delivery to balance oxidant availability against over-oxidation risk.

The difficulty in controlling radical selectivity is further compounded by the fragility of the iron catalysts themselves, as catalyst stability represents a genuinely underreported limitation across the reviewed systems. In cyclen-based homogeneous systems, kinetic analysis confirmed that the coupling product coordinates the iron center, effectively inhibiting the catalytic turnover. Phosphasalen complexes exhibited rapid reduction to a catalytically inactive oxidation state, with chemical rearrangement proceeding on a subsecond time scale. Heterogeneous systems were not exempt, as layered double hydroxide supports underwent progressive structural collapse under reaction conditions, while iron active sites leached gradually from oxide-based supports over repeated cycling, limiting practical operation windows to only a few runs. Coordinating solvents and Lewis basic additives were additionally shown to competitively sequester the iron center. Rational mitigation strategies include stronger chelating ligand designs, covalent immobilization of iron onto support materials, and systematic postcatalysis characterization to identify dominant deactivation pathways in individual systems.

Taken together, the substrate, selectivity, and stability challenges outlined above converge on what is perhaps the most significant unmet goal in this field—the development of genuinely enantioselective iron-catalyzed oxidative coupling. The vast majority of catalyst systems reviewed operate as achiral platforms, producing racemic products despite the pharmaceutical and materials relevance of the chiral scaffolds they generate. Where enantioselective systems were examined, selectivities remained modest, with the best recorded enantiomeric ratios falling short of synthetically useful thresholds. Addressing this gap will require the development of well-defined chiral iron complexes with rigid coordination geometries, deeper mechanistic understanding of the stereo-determining step, and the application of design principles from asymmetric catalysis to iron-centered systems that have yet to be fully explored.

## 5 Perspectives and Future Directions

Building on the limitations identified in the preceding section, this analysis aims to assess the operational parameters that govern reactivity, selectivity, and sustainability across C–C, C–N, C–P, and heterocycle synthesis in order to identify potential research directions that focus on safer reaction conditions, greater environmental compatibility, higher yields with improved selectivity, and the elimination of toxic precursors. In this context, developments in catalyst design, mechanistic understanding, and reaction engineering are examined collectively, with particular attention to how emerging strategies in iron catalysis can address the unresolved challenges that currently limit the broader synthetic applicability of these methods.

Techniques such as the integration of visible light activation, ligand-to-metal charge transfer processes, and cooperative redox networks have emerged as energy-efficient alternatives, allowing oxidative coupling reactions to proceed under increasingly mild and sustainable conditions. Iron salts such as FeCl_3_, FeBr_3_, and Fe­(II)/Fe­(III) complexes are often found to be more effective when used in combination with light, redox mediators, or auxiliary catalysts, suggesting that hybrid activation strategies represent a particularly promising avenue for expanding the substrate scope and selectivity of iron-catalyzed methods.

Rational ligand design spanning phthalocyanine, salen, and macrocyclic frameworks, as well as bioinspired iron systems, has clarified how steric and electronic tuning governs catalyst performance and mechanistic behavior. Building upon these insights, complementary heterogeneous catalysts, including iron oxides, ferrites, single-atom sites, and engineered supports, offer practical advantages in catalyst recovery and efficient oxygen activation under aerobic conditions. In parallel, advances in spectroscopic, electrochemical, and isotopic investigations have sharpened the mechanistic picture of iron-catalyzed oxidative coupling, establishing the foundation upon which more rationally designed and robust catalytic systems can be developed.

Looking ahead, future work is expected to prioritize the development of robust, heterogeneous, and flow-compatible platforms suitable for industrial translation. Advanced mechanistic elucidation through operando spectroscopy, computational modeling, and data-driven optimization is expected to accelerate the rational catalyst design. Taken together, the demonstrated ability of iron catalysts to operate under molecular oxygen activation with inexpensive feedstocks and minimal additives makes these approaches sustainable alternatives to noble metal-based processes in next-generation synthetic chemistry. This review is intended to stimulate further research and promote the broader adoption of more environmentally benign methodologies, continuing to drive the development of greener pathways in synthesis.

## References

[1] Shetgaonkar S. E., Raju A., China H., Takenaga N., Dohi T., Singh F. V. (2022). Non-Palladium-Catalyzed Oxidative Coupling Reactions
Using Hypervalent Iodine Reagents. Front. Chem.

[2] Funes-Ardoiz I., Maseras F. (2018). Oxidative Coupling
Mechanisms: Current State of Understanding. ACS Catal.

[3] Shalit H., Dyadyuk A., Pappo D. (2019). Selective Oxidative Phenol Coupling
by Iron Catalysis. J. Org. Chem.

[4] Dong Y., Xie C., Chen J., Shen A., Luo Q. Q., He B., Wang Z. F., Chang B., Yang F., Shi Z. C. (2022). Iron Catalyzed
C-C Dehydrogenative Coupling Reaction: Synthesis of Arylquinones from
Quinones/Hydroquinones. RSC Adv.

[5] Lu H., Zhou C., Wang Z., Kato T., Liu Y., Maruoka K. (2022). Fe-Catalyzed Three-Component
Coupling Reaction of α,β,γ,δ-Unsaturated
Carbonyl Compounds and Conjugate Dienes with Alkylsilyl Peroxides
and Nucleophiles. J. Org. Chem.

[6] Chen X., Chen T., Ji F., Zhou Y., Yin S. F. (2015). Iron-Catalyzed
Aerobic Oxidative Functionalization of Sp3 C-H Bonds: A Versatile
Strategy for the Construction of N-Heterocycles. Catal. Sci. Technol.

[7] Mintz T., More N. Y., Gaster E., Pappo D. (2021). Iron-Catalyzed Oxidative
Cross-Coupling of Phenols and Tyrosine Derivatives with 3-Alkyloxindoles. J. Org. Chem.

[8] Wencel-Delord J., Glorius F. (2013). C-H Bond Activation Enables the Rapid Construction
and Late-Stage Diversification of Functional Molecules. Nat. Chem.

[9] Evtyugin D. D., Magina S., Evtuguin D. V. (2020). Recent Advances in the Production
and Applications of Ellagic Acid and Its Derivatives. A Review. Molecules.

[10] da
Silva E. M., Vidal H. D. A., Januário M. A. P., Corrêa A. G. (2023). Advances in the Asymmetric Synthesis
of BINOL Derivatives. Molecules.

[11] Killoran P. M., Rossington S. B., Wilkinson J. A., Hadfield J. A. (2016). Expanding the Scope
of the Babler–Dauben Oxidation: 1,3-Oxidative Transposition
of Secondary Allylic Alcohols. Tetrahedron Lett.

[12] Jiang J., Gao Y., Pang S. Y., Lu X. T., Zhou Y., Ma J., Wang Q. (2015). Understanding
the Role of Manganese Dioxide in the Oxidation of Phenolic
Compounds by Aqueous Permanganate. Environ.
Sci. Technol.

[13] Fatykhov R.
F., Khalymbadzha I. A., Sharapov A. D., Potapova A. P., Mochulskaya N. N., Tsmokalyuk A. N., Ivoilova A. V., Mozharovskaia P. N., Santra S., Chupakhin O. N. (2022). MnO2-Mediated Oxidative Cyclization
of “Formal” Schiff’s Bases: Easy Access to Diverse
Naphthofuro-Annulated Triazines. Molecules.

[14] Jalali M., Bissember A. C., Yates B. F., Wengryniuk S. E., Ariafard A. (2021). Oxidation of Electron-Deficient
Phenols Mediated by
Hypervalent Iodine­(V) Reagents: Fundamental Mechanistic Features Revealed
by a Density Functional Theory-Based Investigation. J. Org. Chem.

[15] Yang C., Zhang G., Tang S., Pan Y., Shao H., Jiao W. (2023). Dess–Martin Periodinane-Mediated Oxidative Coupling Reaction
of Isoquinoline with Benzyl Bromide. Molecules.

[16] Sun Y., Abdukader A., Zhang H., Yang W., Liu C. (2017). Copper-Catalyzed
Aerobic Oxidative C-O Bond Formation for the Synthesis of 3,5-Disubstituted
Isoxazoles from Enone Oximes. RSC Adv.

[17] Chang S., Wang J. F., Dong L. L., Wang D., Feng B., Shi Y. T. (2017). Ag­(I)/Persulfate-Catalyzed
Decarboxylative Coupling
of α-Oxocarboxylates with Organotrifluoroborates in Water under
Room Temperature. RSC Adv.

[18] Hossini H., Shafie B., Niri A. D., Nazari M., Esfahlan A. J., Ahmadpour M., Nazmara Z., Ahmadimanesh M., Makhdoumi P., Mirzaei N., Hoseinzadeh E. (2022). A Comprehensive
Review on Human Health Effects of Chromium: Insights on Induced Toxicity. Environ. Sci. Pollut. Res.

[19] Kaur A., Ariafard A. (2020). Mechanistic Investigation
into Phenol Oxidation by
IBX Elucidated by DFT Calculations. Org. Biomol.
Chem.

[20] Watanabe Y., Morozumi H., Mutoh H., Hagiwara K., Inoue M. (2023). Total Synthesis
of (−)-Batrachotoxin Enabled by a Pd/Ag-Promoted Suzuki–Miyaura
Coupling Reaction. Angew. Chem. Int. Ed.

[21] Gavriilidis A., Constantinou A., Hellgardt K., Hii K. K. M., Hutchings G. J., Brett G. L., Kuhn S., Marsden S. P. (2016). Aerobic Oxidations
in Flow: Opportunities for the Fine Chemicals and Pharmaceuticals
Industries. React. Chem. Eng.

[22] Hay A. S., Blanchard H. S., Endres G. F., Eustance J. W. (1959). Polymerization by
Oxidative Coupling. J. Am. Chem. Soc.

[23] Gopalaiah K., Saini A., Chandrudu S. N., Rao D. C., Yadav H., Kumar B. (2017). Copper-Catalyzed Aerobic
Oxidative Coupling of o-Phenylenediamines
with 2-Aryl/Heteroarylethylamines: Direct Access to Construct Quinoxalines. Org. Biomol. Chem.

[24] Chen Z., Vorobyeva E., Mitchell S., Fako E., Ortuño M. A., López N., Collins S. M., Midgley P. A., Richard S., Vilé G., Pérez-Ramírez J. (2018). A Heterogeneous
Single-Atom Palladium Catalyst Surpassing Homogeneous Systems for
Suzuki Coupling. Nat. Nanotechnol.

[25] von
Münchow T., Pandit N. K., Dana S., Boos P., Peters S. E., Boucat J., Liu Y. R., Scheremetjew A., Ackermann L. (2025). Enantioselective C–H Annulations Enabled by
Either Nickel- or Cobalt-Electrocatalysed C–H Activation for
Catalyst-Controlled Chemodivergence. Nat. Catal.

[26] Moselage M., Li J., Ackermann L. (2016). Cobalt-Catalyzed
C-H Activation. ACS Catal.

[27] Zhuang H., Li H., Zhang S., Yin Y., Han F., Sun C., Miao C. (2020). TEMPO and Its Derivatives
Mediated Reactions under Transition-Metal-Free
Conditions. Chin. Chem. Lett.

[28] Hone C. A., Kappe C. O. (2019). The Use of Molecular
Oxygen for Liquid Phase Aerobic
Oxidations in Continuous Flow. Top. Curr. Chem.

[29] Mukherjee A., Cranswick M. A., Chakrabarti M., Paine T. K., Fujisawa K., Münck E., Que L. (2010). Oxygen Activation at Mononuclear
Nonheme Iron Centers: A Superoxo Perspective. Inorg. Chem.

[30] Yu J., Lai W. (2021). Mechanistic Insights
into Dioxygen Activation by a Manganese Corrole
Complex: A Broken-Symmetry DFT Study. RSC Adv.

[31] Wang C. C., Chang H. C., Lai Y. C., Fang H., Li C. C., Hsu H. K., Li Z. Y., Lin T. S., Kuo T. S., Neese F., Ye S., Chiang Y. W., Tsai M. L., Liaw W. F., Lee W. Z. (2016). A Structurally
Characterized Nonheme
Cobalt-Hydroperoxo Complex Derived from Its Superoxo Intermediate
via Hydrogen Atom Abstraction. J. Am. Chem.
Soc.

[32] Guo J., Xie Y., Wu Q. L., Zeng W. T., Chan A. S. C., Weng J., Lu G. (2018). Copper-Catalyzed Aerobic Decarboxylative Coupling between Cyclic
α-Amino Acids and Diverse C-H Nucleophiles with Low Catalyst
Loading. RSC Adv.

[33] Shen Y., Chen F., Du Z., Zhang H., Liu J., Liu N. (2024). Cu­(I) Complexes Catalyzed
the Dehydrogenation of N-Heterocycles. J. Org.
Chem.

[34] Sahu S., Goldberg D. P. (2016). Activation of Dioxygen by Iron and Manganese Complexes:
A Heme and Nonheme Perspective. J. Am. Chem.
Soc.

[35] Solomon E. I., Goudarzi S., Sutherlin K. D. (2016). O_2_ Activation by Non-Heme
Iron Enzymes. Biochemistry.

[36] Darari M., Francés-Monerris A., Marekha B., Doudouh A., Wenger E., Monari A., Haacke S., Gros P. C. (2020). Towards
Iron­(II) Complexes with Octahedral Geometry: Synthesis, Structure
and Photophysical Properties. Molecules.

[37] Zhu Y. Y., Li H. Q., Ding Z. Y., Lü X. J., Zhao L., Meng Y. S., Liu T., Gao S. (2016). Spin Transitions
in a Series of [Fe­(Pybox)_2_]^2+^ Complexes Modulated
by Ligand Structures, Counter Anions, and Solvents. Inorg. Chem. Front.

[38] Chábera P., Kjaer K. S., Prakash O., Honarfar A., Liu Y., Fredin L. A., Harlang T. C. B., Lidin S., Uhlig J., Sundström V., Lomoth R., Persson P., Wärnmark K. (2018). FeII Hexa
N-Heterocyclic Carbene Complex with a 528 ps Metal-To-Ligand Charge-Transfer
Excited-State Lifetime. J. Phys. Chem. Lett.

[39] Gordon J. B., Albert T., Dey A., Sabuncu S., Siegler M. A., Bill E., Moënne-Loccoz P., Goldberg D. P. (2021). A Reactive,
Photogenerated High-Spin (S = 2) FeIV­(O) Complex via O_2_ Activation. J. Am. Chem. Soc.

[40] Lee N. Y., Mandal D., Bae S. H., Seo M. S., Lee Y. M., Shaik S., Cho K. B., Nam W. (2017). Structure and Spin
State of Nonheme FeIVO Complexes Depending on Temperature: Predictive
Insights from DFT Calculations and Experiments. Chem. Sci.

[41] England J., Guo Y., Van Heuvelen K. M., Cranswick M. A., Rohde G. T., Bominaar E. L., Münck E., Que L. (2011). A More Reactive Trigonal-Bipyramidal High-Spin Oxoiron­(IV) Complex
with a Cis-Labile Site. J. Am. Chem. Soc.

[42] Bigi J. P., Harman W. H., Lassalle-Kaiser B., Robles D. M., Stich T. A., Yano J., Britt R. D., Chang C. J. (2012). A High-Spin Iron­(IV)-Oxo
Complex Supported by a Trigonal Nonheme Pyrrolide Platform. J. Am. Chem. Soc.

[43] Wandzilak A., Grubel K., Skubi K. L., McWilliams S. F., Bessas D., Rana A., Hugenbruch S., Dey A., Holland P. L., DeBeer S. (2023). Mössbauer and Nuclear Resonance
Vibrational Spectroscopy Studies of Iron Species Involved in N-N Bond
Cleavage. Inorg. Chem.

[44] Delanoy G., Lupardus C., Vali S. W., Wofford J. D., Thorat S., Lindahl P. A. (2024). Mössbauer
and EPR Detection of Iron Trafficking
Kinetics and Possibly Labile Iron Pools in Whole Saccharomyces Cerevisiae
Cells. J. Biol. Chem.

[45] Solomon E. I., Wong S. D., Liu L. V., Decker A., Chow M. S. (2009). Peroxo
and Oxo Intermediates in Mononuclear Nonheme Iron Enzymes and Related
Active Sites. Curr. Opin. Chem. Biol.

[46] Purtsas A., Rosenkranz M., Dmitrieva E., Kataeva O., Knölker H.-J. (2022). Iron-Catalyzed
Oxidative C–O and C–N Coupling Reactions Using Air as
Sole Oxidant. Chem. - Eur. J.

[47] Wusiman A., Hudabaierdi R. (2019). Iron-Catalyzed Aerobic Oxidative Cross-Coupling Amidation
of N,N-Dimethylanilines. Tetrahedron Lett.

[48] Parmeggiani C., Cardona F. (2012). Transition Metal Based
Catalysts in the Aerobic Oxidation
of Alcohols. Green Chem.

[49] Purtsas A., Kataeva O., Knölker H.-J. (2020). Iron-Catalyzed
Oxidative C–C
Cross-Coupling Reaction of Tertiary Anilines with Hydroxyarenes by
Using Air as Sole Oxidant. Chem. - Eur. J.

[50] Brezny A. C., Lyke J. H., Olsen S. E., Straton E. R., Zubarieva O. (2025). Mechanism
of Catalyst Activation in Iron­(Porphyrin)-Catalysed Aerobic Oxidative
Cleavage of 2,3-Dimethylindole. Dalton Trans.

[51] Hu P., Tan M., Cheng L., Zhao H., Feng R., Gu W. J., Han W. (2019). Bio-Inspired
Iron-Catalyzed Oxidation of Alkylarenes Enables Late-Stage
Oxidation of Complex Methylarenes to Arylaldehydes. Nat. Commun.

[52] Fonseca A., Marquez C., De Vos D. (2026). Iodine/Iron
Oxide-Catalysed Aerobic
Oxidative Coupling of Phosphites and Alcohols via in Situ Iodophosphate
Formation. Chem. Commun.

[53] Miao C., Zhao H., Zhao Q., Xia C., Sun W. (2016). NHPI and Ferric
Nitrate: A Mild and Selective System for Aerobic Oxidation of Benzylic
Methylenes. Catal. Sci. Technol.

[54] Sharma A., Kour H., Kour J., Kamal N., Sawant S. D. (2022). Visible-Light-Promoted
Iron-Catalyzed C-H Functionalization of 1,4-Naphthoquinones via Oxidative
Coupling with Sulfoximines. Chem. Commun.

[55] Yang Y., Yu X., He N., Huang X., Song X., Chen J., Lin J., Jin Y. (2023). FeCl_3_-Catalyzed Oxidative Amidation of Benzylic
C-H Bonds Enabled by a Photogenerated Chlorine-Radical. Chem. Commun.

[56] Singh M., Prasad P. R. (2025). Green and Efficient Iron-Catalyzed Cross-Dehydrogenative
Coupling for the Synthesis of α,β-Unsaturated Ketones
via C­(sp^3^)-H Functionalization. RSC
Adv.

[57] Wu K. X., Xu Y. Z., Cheng L., Wu R. S., Liu P. Z., Xu D. Z. (2021). Iron-Catalyzed Oxidative
Bis-Arylation of Indolin-2-Ones for Direct
Construction of Quaternary Carbons. Green Chem.

[58] Wu H.-R., Huang H.-Y., Ren C.-L., Liu L., Wang D., Li C.-J. (2015). FeIII-Catalyzed Cross-Dehydrogenative
Arylation (CDA) between Oxindoles
and Arenes under an Air Atmosphere. Chem. -
Eur. J.

[59] Kumar N. V. A., Kamath S., Gaonkar S. L., Shetty N. S. (2016). An Aerobic Oxidative
Coupling Approach for the Synthesis of N-Substituted 2-Aminobenzothiazole
Derivatives Using Iron Catalyst. Orient. J.
Chem.

[60] Cai J., Liu Y., Jiang Y., Yang Y. (2017). Iron-Catalyzed Aerobic Oxidative
Phosphonation of N-Aryl Tetrahydroisoquinolines. Phosphorus, Sulfur Silicon Relat. Elem.

[61] Hu R., Han D., Li N., Huang J., Feng Y., Xu D. (2020). Iron-Catalyzed
Direct Oxidative Alkylation and Hydroxylation of Indolin-2-ones with
Alkyl-Substituted *N*-Heteroarenes. Angew. Chem.

[62] Wu L. Y., Usman M., Liu W. B. (2020). Enantioselective Iron/Bisquinolyldiamine
Ligand-catalyzed Oxidative Coupling Reaction of 2-naphthols. Molecules.

[63] Fritsche R. F., Theumer G., Kataeva O., Knölker H. (2017). Iron-Catalyzed
Oxidative C–C and N–N Coupling of Diarylamines and Synthesis
of Spiroacridines. Angew. Chem.

[64] Oheix E., Herrero C., Moutet J., Rebilly J.-N., Cordier M., Guillot R., Bourcier S., Banse F., Sénéchal-David K., Auffrant A. (2020). FeIII and FeII Phosphasalen Complexes: Synthesis, Characterization,
and Catalytic Application for 2-Naphthol Oxidative Coupling. Chem. - Eur. J.

[65] Vershinin V., Feruz L. N., Forkosh H., Kertzman L., Libman A., Burés J., Pappo D. (2024). Aerobic Oxidative Coupling of 2-Aminonaphthalenes
by Homogenous Nonheme Iron Catalysts. ACS Catal.

[66] Zhang X., Yang T. M., Hu L. M., Hu X. H. (2022). Stereoselective
Iron-Catalyzed Alkylation of Enamides with Cyclopropanols via Oxidative
C­(sp^2^)-H Functionalization. Org.
Lett.

[67] Tkachenko N. V., Lyakin O. Y., Samsonenko D. G., Talsi E. P., Bryliakov K. P. (2018). Highly
Efficient Asymmetric Aerobic Oxidative Coupling of 2-Naphthols in
the Presence of Bioinspired Iron Aminopyridine Complexes. Catal. Commun.

[68] Geng L., Song J., Zheng B., Wu S., Zhang W., Jia M., Liu G. (2016). Aerobic Oxidative Coupling
of Alcohols and Amines to
Imines over Iron Catalysts Supported on Mesoporous Carbon. Chin. J. Catal.

[69] Chen Z., Song J., Peng X., Xi S., Liu J., Zhou W., Li R., Ge R., Liu C., Xu H., Zhao X., Li H., Zhou X., Wang L., Li X., Zhong L., Rykov A. I., Wang J., Koh M. J., Loh K. P. (2021). Iron Single Atom Catalyzed Quinoline Synthesis. Adv. Mater.

[70] Xia Z., Qin L., Zhou W., Wang H., Yu B., Sun Z., Qian J., He M. (2019). An Efficient Aerobic Oxidative Phosphonation
of α-Amino C-H Bonds over CoNiFe Hydrotalcite. Tetrahedron Lett.

[71] Shaker M., Elhamifar D. (2020). Magnetic Methylene-Based Mesoporous Organosilica Composite-Supported
IL/Pd: A Powerful and Highly Recoverable Catalyst for Oxidative Coupling
of Phenols and Naphthols. Mater. Today Chem.

[72] Nguyen O. T. K., Ha P. T., Dang H. V., Vo Y. H., Nguyen T. T., Le N. T. H., Phan N. T. S. (2019). Superparamagnetic
Nanoparticle-Catalyzed
Coupling of 2-Amino Pyridines/Pyrimidines with trans-Chalcones. RSC Adv.

[73] Baruah M. J., Bora T. J., Dutta R., Roy S., Guha A. K., Bania K. K. (2021). Fe­(III) Superoxide Radicals in Halloysite Nanotubes
for Visible-Light-Assisted Benzyl Alcohol Oxidation and Oxidative
C-C Coupling of 2-Naphthol. Mol. Catal.

[74] Dey A., Hajra A. (2017). FeCl_3_/ZnI_2_-Catalyzed Regioselective Synthesis
of Angularly Fused Furans. Org. Biomol. Chem.

[75] Zhang Y., Yang D., Li Y., Zhao X., Wang B., Qu J. (2019). Biomimetic Catalytic
Oxidative Coupling of Thiols Using Thiolate-Bridged
Dinuclear Metal Complexes Containing Iron in Water under Mild Conditions. Catal. Sci. Technol.

[76] Zhang Z., Ma Y., Dai S., Li L., Zhang Y., Li H. (2020). Iron/TEMPO-Catalyzed
Direct Aerobic Oxidative Coupling of Methyl-Substituted *N*-Heteroazaarenes with Alcohols. Tetrahedron
Lett.

[77] Kumar A., Kumar P., Joshi C., Ponnada S., Pathak A. K., Ali A., Sreedhar B., Jain S. L. (2016). A [Fe­(Bpy)_3_]^2+^ Grafted Graphitic
Carbon Nitride Hybrid for Visible Light Assisted
Oxidative Coupling of Benzylamines under Mild Reaction Conditions. Green Chem.

[78] Zhai Y., Zhang M., Fang H., Ru S., Yu H., Zhao W., Wei Y. (2018). An Efficient Protocol for the Preparation
of Aldehydes/Ketones and Imines by an Inorganic-Ligand Supported Iron
Catalyst. Org. Chem. Front.

